# Formulation, General Features and Global Calibration of a Bioenergetically-Constrained Fishery Model

**DOI:** 10.1371/journal.pone.0169763

**Published:** 2017-01-19

**Authors:** David A. Carozza, Daniele Bianchi, Eric D. Galbraith

**Affiliations:** Department of Earth and Planetary Sciences, McGill University, Montreal, Quebec, Canada; Centro de Investigacion Cientifica y de Educacion Superior de Ensenada Division de Fisica Aplicada, MEXICO

## Abstract

Human exploitation of marine resources is profoundly altering marine ecosystems, while climate change is expected to further impact commercially-harvested fish and other species. Although the global fishery is a highly complex system with many unpredictable aspects, the bioenergetic limits on fish production and the response of fishing effort to profit are both relatively tractable, and are sure to play important roles. Here we describe a generalized, coupled biological-economic model of the global marine fishery that represents both of these aspects in a unified framework, the BiOeconomic mArine Trophic Size-spectrum (BOATS) model. BOATS predicts fish production according to size spectra as a function of net primary production and temperature, and dynamically determines harvest spectra from the biomass density and interactive, prognostic fishing effort. Within this framework, the equilibrium fish biomass is determined by the economic forcings of catchability, ex-vessel price and cost per unit effort, while the peak harvest depends on the ecosystem parameters. Comparison of a large ensemble of idealized simulations with observational databases, focusing on historical biomass and peak harvests, allows us to narrow the range of several uncertain ecosystem parameters, rule out most parameter combinations, and select an optimal ensemble of model variants. Compared to the prior distributions, model variants with lower values of the mortality rate, trophic efficiency, and allometric constant agree better with observations. For most acceptable parameter combinations, natural mortality rates are more strongly affected by temperature than growth rates, suggesting different sensitivities of these processes to climate change. These results highlight the utility of adopting large-scale, aggregated data constraints to reduce model parameter uncertainties and to better predict the response of fisheries to human behaviour and climate change.

## Introduction

Global oceanic wild fish harvest grew at a tremendous rate over the 20th century, increasing by approximately a factor of four between 1950 and 1990 [1]. As a result, biomass has been depleted relative to its pristine state [2, 3], altering ecosystem structures worldwide, with collapses documented in between 7 and 25% of fisheries [4, 5]. Anthropogenic climate change is altering primary production and temperature distributions worldwide [6], with impacts on the ranges of various marine species [7, 8], and on their growth, mortality, reproduction, and recruitment rates [9, 10]. Meanwhile, rapid and ongoing changes in fishing technology [11], the demand for fish products [12], and regulation frameworks are driving changes in the distribution and intensity of fishing effort. Given future projections of increasing human population, and consequent pressure on food resources, it is critical to develop improved predictive understanding of how fisheries will evolve with interacting environmental and human drivers.

Numerical models of fishery-human interactions that can be coupled to representations of the environment, often called end-to-end models, have been extremely helpful to shed light on these issues [13, 14]. However, most of these models are designed primarily in order to study internal ecosystem dynamics, and often include complex parameterizations that are challenging to quantify, such as feeding relationships [15]. In addition, most of these models are designed to represent the present-day community in a specific region, and therefore lack the generality and flexibility required to represent long-term structural changes such as those caused by new invasive species, climate-driven range shifts [7], or fishing-driven evolution [16]. The incomplete view provided by a patchwork of regional models also hampers studies of global food security, given the development of globally interwoven fishing fleets and fish-trading since the 1950’s.

Alternative ecological models that are structurally simpler, founded on macroecological principles, and appropriate for a global range of conditions, have recently begun to allow the direct prediction of fish biomass from environmental variables [17–23]. However, most of these models were designed to study fish, rather than fishing. As a result, fish harvests are generally prescribed in these models, rather than treating the fishing effort as an interactive component, which limits the consideration of economic factors such as changes in fishing technology, market conditions, and regulatory regimes. Furthermore, an extensive exploration of parameter uncertainty is challenging for most of these models due to complexity and/or computational cost, and the simulated fish species may not be equivalent to those harvested, making direct comparisons with observed historical harvest records difficult.

Here we describe the economic component and general characteristics of the BiOeconomic mArine Trophic Size-spectrum model (BOATS), a computationally-efficient model which directly couples a size spectrum model of fish biomass with an interactive economic model on a spatially-resolved global grid. The ecological model, which is described in detail in [24], focuses on a small number of robust bioenergetic principles of marine ecosystems that are of direct relevance for fisheries and environmental change, predicting the growth of all commercially-harvested marine consumers, which we refer to as ‘fish’. The simulated fish spectrum is coupled directly to a prognostic model of fishing effort, at the same grid scale, that evolves over time in response to the local bioeconomically-determined profit. Because BOATS was designed to include only fundamental processes and relatively few parameters, parameter uncertainty can be addressed in a robust and comprehensive way by using a Monte Carlo approach. As discussed below, this allows us to select global parameter combinations that agree with observations at the scale of the Large Marine Ecosystems (LMEs), rather than being locally-tuned. We show that, after optimization, the ensemble has a high degree of skill, making it suitable for studies of first-order interactions between climate, the marine ecosystem and humans. We also point out the most important implications of the remaining parameter uncertainty.

## Materials and Methods

### Model description

#### Overview

The guiding principles during the development of the BiOeconomic mArine Trophic Size-spectrum (BOATS) model were: 1. To maintain general applicability to a broad range of marine ecosystems and fisheries; 2. To include sufficient ecological complexity to represent the fundamental dynamics of commercial fish biomass production, but remain simple enough to be easily interpretable; 3. To minimize the number of uncertain parameters; 4. To include a dynamical representation of fishing activity in the same framework as the ecosystem; 5. To enable long-term projections of the coupled natural-social system with robust and simple external forcings.

**Fig 1:**
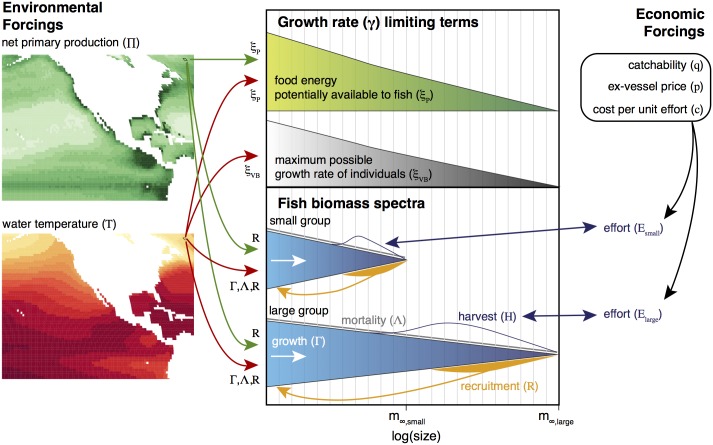
Schematic diagram of the main modules, components, and processes of the BOATS model.

Fig 1 gives a schematic representation of the model. Biomass production is determined by the energy supplied from photosynthesis [25], the life history of individuals according to temperature-dependent growth and mortality rates [26, 27], and stock-productivity-dependent recruitment [28]. Feeding relationships are not explicit, a simplifying assumption motivated by the diversity of marine life and predatory strategies, and the heterogeneity of species inter-relationships in different regions. Life history is resolved in terms of growth from juveniles to reproductively-mature individuals, through a continuous spectrum of size.

The model is deterministic, discretized on a 2-dimensional spatial grid (representing the vertically-integrated ecosystem), and steps forward in time from a set of initial conditions. The model can include an arbitrary number of size spectra, each of which represents a ‘super-organism’ that exists in all grid cells at all times. There is no simulation of horizontal movement by fish or fishing boats, so that each cell is independent (that is, no interaction with other cells). Although this ignores the life history aspects of larval transport and migration, such as important environmental contrasts between spawning grounds and adult feeding grounds [29], these were determined to be difficult problems when considering all types of commercial species, and resolving movement would incur a large computational burden [21]. Fishing effort is interactively determined in each grid cell, and evolves over time as a function of the harvest in that grid cell, dependent on globally-constant economic forcings. In the following, we refer to globally-constant scalars that determine the behavior of a model but do not vary during or between simulations as ‘parameters’, and to a model with a particular set of parameters as a ‘model variant’. Prescribed quantities that influence the model behaviour and may vary during or between simulations are referred to as ‘forcings’ (which may be globally homogenous, or locally variable), while model state variables are referred to as ‘variables’. Key assumptions are summarized in Table 1.

**Table 1:** Key model assumptions.

	Essential	Present version (could be changed)
**Ecosystem**		
	Mass is the ecological organizing variable	Community represented by 3 spectra with different asymptotic sizes
	Biomass individual flux follows the McKendrick-von Foerster equation	No movement of fish between grid cells
	Asymptotic size is the spectrum-defining trait	Implicit feeding relationships
	Production dependent on NPP, T, and stock-dependent recruitment	Fish spawn continuously
	Individual fish growth cannot exceed a physiological upper limit	Potentially-commercial species aggregated by size (finfish and invertebrates)
**Economics**		
	Fishing effort is the fundamental economic variable	Open access (no management)
	Technology determines catchability	Local effort varies with the profitability of fishing at that point
	Effort and biomass determine harvest through catchability	Harvest is linear in effort and biomass
	Selectivity function determines the size-structure of harvest	No movement of fishing vessels between grid cells
		Fleet dynamics parameter is globally constant
		Cost per unit effort is globally constant
		Price is globally constant, and does not vary by group or size
		Catchability is globally constant, but is varied over time

Below, we elaborate on details of the model. First, we present a brief overview of the ecosystem module formulation and equations, and refer the reader to [24] for a full description. Second, we describe the dynamic determination of fishing effort. Third, we discuss the allocation of effort over the size spectra, representing the selectivity of fishing gear.

#### Ecological module

BOATS resolves an arbitrary number of size spectra that are defined by their asymptotic sizes and, together, are defined as the sum of all consumers (including both finfish and invertebrates) that were commercially harvested before the year 2006. Here we discretize the commercial species among three spectra, which we refer to as small, medium, and large groups, referring to their asymptotic size.

The evolution of biomass in each group spectrum is represented using the McKendrick-von Foerster model [30, 31],

$$\begin{array}{cc} \frac{\partial }{\partial t}f_{k} & =-\frac{\partial }{\partial m}\gamma_{k}f_{k}+\frac{\gamma_{k}f_{k}}{m}-\Lambda_{k}f_{k}, \end{array}$$ (1)

where *m* is the individual fish mass, *t* is time, *f*_*k*_ (gwB m^−2^ g^−1^, where gwB are grams of wet biomass) is the biomass spectrum of group *k*, *γ*_*k*_ (g s^−1^) the group growth rate, and Λ_*k*_ (s^−1^) the group natural mortality rate.

Fish biomass growth rates are limited by a production spectrum that estimates the energy (here assumed to be equivalent to biomass) that can be provided to any given size through trophic transfer from primary production *ξ*_*P*,*k*_ (g s^−1^) [18, 25, 32], and are also limited by allometric estimates of the rates at which individual fish can grow *ξ*_*VB*,*k*_ (g s^−1^) [28, 33, 34]. Underlying the simulated biomass spectra are the calculated flows of biomass energy from photosynthesis to fish via the trophic transfer, which we express as

$$\begin{array}{c} \xi_{P,k}=\frac{\phi_{C,k}\pi m}{f_{k}}, \end{array}$$ (2)

where *π* (gwB m^−2^ g^−1^ s^−1^) is the spectrum of energy from primary production available to each individual of size *m* that depends on the trophic scaling *τ* as the exponent of a power law dependence, and *ϕ*_*C*,*k*_ is the fraction of primary production allocated to a commercial group *k* [18, 25, 32]. Furthermore, we assume that the flow of biomass energy from small to large fish, which occurs as individuals grow according to empirical allometric rules, cannot exceed a maximum physiological rate

$$\begin{array}{c} \xi_{VB,k}=Am^{b}-k_{a}m, \end{array}$$ (3)

where *b* (unitless) is the allometric scaling constant, *A* (g s^−1^) is the allometric growth rate, and *k*_*a*_ (s^−1^) is the mass specific investment in activity [28, 33, 34]. The allometric growth rate *A* is the product of a temperature-independent rate *A*_0_ (g^1−b^ s^−1^) and a van’t Hoff–Arrhenius (exponential) temperature dependence *a*_*A*_(*T*) which is set by the growth activation energy of metabolism *ω*_*a*,*A*_ (eV) parameter [25].

The realized input energy to growth and reproduction in each size class, *ξ*_*I*_ (g s^−1^), is the minimum of *ξ*_*P*_ and *ξ*_*VB*_. This implies that, given an excess of food, individual fish will grow as fast as biologically possible, given their temperature-dependent metabolism. If, instead, the production of food at a given location is insufficient to fully feed the existing biomass, this production will be equally partitioned among all individuals and will limit growth rates. When there is no harvest applied, primary production, and thus *ξ*_*P*_, generally limits growth over the biomass spectrum.

The natural mortality rate Λ_*k*_ is based on an empirical relationship that depends on the individual and asymptotic mass, and is first order in fish biomass, according to

$$\begin{array}{c} \Lambda_{k}=\lambda m^{-h}m_{\infty ,k}^{h+b-1}, \end{array}$$ (4)

where *h* is the mortality allometric scaling and *m*_∞,*k*_ is the asymptotic mass of group *k* [26, 27]. The component of the mortality rate that is independent of mass, *λ*, is proportional to $e^{\zeta_{1}}A_{0}a_{\lambda }\left(T\right)$, where *ζ*_1_ is the mortality rate parameter and *a*_*λ*_(*T*) the temperature dependence of mortality, which, as with growth, takes the van’t Hoff–Arrhenius functional form and is set by the mortality activation energy of metabolism parameter *ω*_*a*,*λ*_ (eV).

Recruitment forms the bottom boundary condition to Eq (1), and is written as

$$\begin{array}{c} f_{k}\left(m_{0}\right)\gamma_{k}\left(m_{0}\right)=R_{P,k}\frac{R_{e,k}}{R_{P,k}+R_{e,k}}. \end{array}$$ (5)

where *R*_*e*,*k*_ is the potential generation of new recruits from egg production, itself a function of 1. the primary production and the fish biomass that is able to reproduce and 2. the fraction of larvae that survive to the recruit size *m*_0_ [34], whereas *R*_*P*,*k*_ is the potential flux of primary production input to juvenile fish.

The model is forced with two-dimensional, global, monthly climatological fields of net primary production and temperature (S1 and S2 Figs). A schematic of BOATS, illustrating the main modules, components, and processes, is presented in Fig 1, and the full set of biological model parameters are presented in S1 Table.

#### The determination of fishing effort

Harvest depends upon fish biomass and the fishing effort. Here, we interpret effort as the combined inputs used in the fishing process [35], essentially fuel, labour and the construction and maintenance of fishing fleets, and we express it in energy units of W m^−2^ (1 W = 1 J s^−1^). This choice facilitates comparison with the global effort database of [1, 36]. Effort of this type is often described as ‘nominal effort’, and is defined here as being independent of fishing technology.

The allocation of fishing effort, that is, the dynamics of fleets of fishing vessels, involves biological, social, and economic aspects that depend on numerous variables that operate on a variety of timescales [37, 38]. In order to provide a first step in prognostic modeling of these interactions in a simple yet robust way, we couple the Gordon-Schaeffer open-access fisheries economics model [39, 40] to our ecological model [24] in a discrete, temporally-resolved framework. This open-access model assumes an absence of regulation, so that fishermen tend to individually seek the greatest total harvest [39, 41], which ultimately leads to overharvest and to the tragedy of the commons [42, 43]. Although this is not an economically or biologically optimal outcome, it is common, given the difficulty in establishing property rights over living marine resources. Although management has made great advances in effectively counteracting this tendency in some fisheries, particularly since the 1990s [44, 45], the open-access result has historically dominated, and remains a cause of overfishing in many open-ocean and coastal fisheries [46]. Subsidies can also shift behaviour from the open-access solution, although wherever subsidies are capacity-building they often exacerbate the open-access problem [47].

In open-access fisheries, fishermen seek to maximize their individual harvests, and so are driven by average instead of marginal profit [39]. Effort in a fish group *k*, *E*_*k*_(*t*) (W m^−2^), therefore evolves in time as a linear function of the average profit (profit per unit effort), as

$$\begin{array}{c} \frac{d}{\text{dt}}E_{k}\left(t\right)=\kappa_{e}{\left[\text{average}\;\text{profit}\right]}_{k}=\frac{\kappa_{e}\left[{\text{revenue}}_{k}-{\text{cost}}_{k}\right]}{E_{k}\left(t\right)}, \end{array}$$ (6)

where *κ*_*e*_ (W^2^ m^−2^ $^−1^) is the fleet dynamics parameter [48] that determines the timescale at which a change in average profit affects effort, and revenue and cost are in units of $ m^−2^ s^−1^. The fleet dynamics parameter *κ*_*e*_ is set so that the adjustment of effort to changes in profit is approximately 10 years, since fleets adjust to changes in biomass on roughly decadal timescales [49–51]. Although the entry of effort into fisheries often occurs at a faster rate than exit, particularly when subsidies are applied, for simplicity we assume here that both rates are the same.

We assume that harvest is linear in both biomass and effort, and so for a given level of harvesting technology, a fixed amount of effort always catches the same fraction of biomass [40]. The ecological model discretizes biomass along a size spectrum, and so we define an analagous harvest spectrum. The harvest *h*_*k*_ (gwB m^−2^ s^−1^ g^−1^) in the mass range d*m* is that which results from effort *E*_*k*_ applied to group *k* during time interval d*t*, which we define as

$$\begin{array}{c} h_{k}dt\;dm=q_{k}\sigma_{k}E_{k}f_{k}dt\;dm, \end{array}$$ (7)

where *q*_*k*_ (m^2^ W^−1^ s^−1^) is the catchability and *σ*_*k*_ (unitless) is the size-dependent selectivity of harvest on group *k* (discussed further in the following subsection).

The catchability *q*_*k*_ of fish in group *k* is the fraction of selectable biomass harvested for a unit amount of effort [52]. The catchability encapsulates the ease with which fish can be harvested given the inherent characteristics of fish, and the level of applied technology, including both embodied and disembodied aspects of technology [11, 53]. Thus, the adoption of improved vessels and fishing gear, sonar, communications, and navigation technologies, as well as improved skipper knowledge and efficiency practices, would all cause an increase of *q*_*k*_. In contrast, *q*_*k*_ does not include technological gains that reduce the cost of fishing per unit effort. Nor does *q*_*k*_ include technological gains that might enhance the market value of fish or provide access to new markets, which would increase the ex-vessel price.

The revenue spectrum *r*_*k*_ ($ m^−2^ s^−1^ g^−1^) is the ex-vessel price *p*_*k*_ ($ gwB^−1^, the price that fishermen are paid at port) of the harvest multiplied by the harvest spectrum *h*_*k*_ of Eq (7), and so *r*_*k*_ dtdm gives the revenue associated with the harvest of group *k* for fish of mass range d*m* at time *t* during d*t*,

$$\begin{array}{c} r_{k}dt\;dm=p_{k}q_{k}\sigma_{k}E_{k}f_{k}dt\;dm. \end{array}$$ (8)

Studies have shown that when numerous species are considered together, a scale that conceptually matches our approach, the price is not strongly related to the trophic level [54]. Further, given the unpredictability of fish prices as a function of size, which relates to complex factors such as transportation, processing and societal preferences, we presently assume that the ex-vessel price is globally constant.

The revenue component of the change in effort expressed in Eq (6) is therefore calculated by integrating over mass, so that

$$\begin{array}{c} {\text{revenue}}_{k}=\int_{m_{0}}^{m_{\infty ,k}}r_{k}dm\;dt=q_{k}E_{k}dt\int_{m_{0}}^{m_{\infty ,k}}p_{k}\sigma_{k}f_{k}dm. \end{array}$$ (9)

Note that, although the harvest is resolved as a size spectrum, there is only a single scalar value of effort for a given fish group *k* at a given location and time, since it is assumed that the fishing effort does not discriminate among size within each group, other than as determined by the gear type through the selectivity function.

We assume that cost is proportional to effort [37], such that

$$\begin{array}{c} C_{k}dt=c_{k}E_{k}dt, \end{array}$$ (10)

where *C*_*k*_ is the cost ($ m^−2^ s^−1^) associated with the effort exerted on fishing group *k*, and *c*_*k*_ ($ W^−1^ s^−1^) is the cost per unit effort. We estimate the global cost per unit effort forcing by assuming that the real world was close to an open-access equilibrium between 1990 and 2006, so that total global revenue was equal to the total global cost, which is a reasonable first-order assumption based on the fishing cost database of Lam et al., [55] (see Table 5 of that article). Thus, we divide the global revenue from the SAUP harvest database by the global effort as reported in [1]. We presently assume that cost has no spatial dependence, for simplicity. Although this ignores the potentially-important role of the transit distance between fishing grounds and ports, it avoids a great deal of complexity, and could be revisited in future work.

#### Linking effort to harvest: harvest selectivity

The size selectivity curve *σ*_*k*_(*m*) plays a fundamental role in determining the size spectrum of fish biomass and harvest [56–59]. A variety of functional forms have been used to describe the selectivity, all of which avoid the smallest sizes, and can be generalized as either dome-shaped or sigmoidal. Dome-shaped curves are used for selective gears such as gillnets, driftnets, and longlines, which are designed to avoid larger organisms that belong to non-targeted species. Sigmoidal curves are used for towed gears such as trawls and seines, as well as traps and dredges, that do not discriminate against large organisms [56]. Given that between 65% [55] and 75% [60, 61] of global harvest is associated with sigmoidal selectivity gear types (S2 Table), and because the modeled effort is by construction perfectly selective by group, perfectly avoiding the harvest of larger organism types, a sigmoidal form is most appropriate. We adapt the formulation of [34],

$$\begin{array}{c} \sigma_{k}={\left[1+{\left(\frac{m}{m_{\Theta ,k}}\right)}^{-c_{\sigma }/\delta_{2}}\right]}^{-1}, \end{array}$$ (11)

where *m*_Θ,*k*_ is the threshold mass of fish in group *k*, and *c*_*σ*_ is the selectivity slope, which we assume takes the same constant value over all groups (S3 Fig). We interpret the selectivity to be sigmoidal in length due to the physical constraints of nets [57], and therefore convert to mass using the mass-length relationship $m=\delta_{1}l^{\delta_{2}}$, where *m* and *l* are fish mass and length, respectively, and *δ*_1_ and *δ*_2_ are constants [62].

The threshold mass of fish for a group is defined in terms of the maturity mass *m*_*α*,*k*_ such that *m*_Θ,*k*_ = *d*_*m*_Θ_,*k*_*e*_*m*_Θ_,*k*_*m*_*α*,*k*_. By means of the parameter *d*_*m*_Θ_,*k*_, we adjust the threshold mass of the large group to 25% of the maturity mass, that of the middle group to 50% of the maturity mass, and leave the threshold mass of the small group equal to the maturity mass. This could be thought of as representing proportionally greater bycatch of the juveniles of larger fish species. The parameter *e*_*m*_Θ_,*k*_ is used solely to test uncertainty in the threshold mass. Table 2 lists the economic model forcings and variables.

**Table 2:** Economic forcings, parameters and variables.

		Name	Value [Range]	Unit	Eq	Reference
**Forcings**						
	*q*_*k*_(*t*)	Catchability	-	m^2^ W^−1^ s^−1^	Eq (7)	[40]
	*p*_*k*_	Ex-vessel price	-	$ gwB^−1^	Eq (8)	[37]
	*c*_*k*_	Cost per unit effort	-	$ W^−1^ s^−1^	Eq (10)	[37]
**Parameters**						
	*κ*_*e*_	Fleet dynamics parameter	10^−6^	W $^−1^ s^−1^	Eq (6)	[51]
	*c*_*σ*_	Slope of *σ*_*k*_(*m*)	16.7787 [12, 24]	Unitless	Eq (11)	[34]
	*d*_*m*_Θ,*k*__	Selectivity mass adjustment	(1 0.5 0.25)	Unitless	Eq (11)	-
	*e*_*m*_Θ,*k*__	Selectivity mass scaling	0.6198 [0.5, 1.5]	Unitless	Eq (11)	-
	*σ*_*k*_(*m*)	Selectivity function	-	Unitless	Eqs (7) and (11)	[56]
**Variables**						
	*h*_*k*_(*m*, *t*)	Harvest spectrum	-	gwB m^−2^s^−1^g^−1^	Eq (7)	-
	*E*_*k*_(*t*)	Fishing effort	-	Wm^−2^	Eq (7)	[40]
	*r*_*k*_(*m*, *t*)	Revenue spectrum	-	$ m^−2^s^−1^g^−1^	Eq (8)	-
	*C*_*k*_(*t*)	Total cost	-	$ m^−2^ s^−1^	Eq (10)	[37]

### Assessment of parameter uncertainty and calibration

In order to evaluate uncertain model parameter values, we compare model simulations with two primary sources of global data. The first is stock assessment data as compiled in the RAM Legacy database [63]. These data provide an estimate of fish biomass, which is extremely useful; however, for most ecosystems, the stock assessments do not include the entirety of harvested species, limiting their comparability to BOATS. Therefore, we also make use of global catch data, described in greater detail below, as reconstructed by the Sea Around Us Project (SAUP) [60, 61].

Because the harvest depends on economic as well as ecological forcings, there are multiple economic and ecological combinations that can produce the same harvest in a given region. On its own, this could lead to an intractable problem, with too many degrees of freedom. However, we can take advantage of the fact that there is a maximum rate of fish production that an ecosystem can achieve at any given location. Because of the historical increase of fishing intensity, most ecosystems are presently either close to this peak value (fully exploited), or have passed it and gone into decline (over-exploited) or collapse [64]. We identify the observed maximum multi-year harvest for each LME (which we denote as ${}_{l}H_{\text{LMEmax}}^{\text{obs}}$, where *l* = 1, …, 66 is an index for the LMEs) as an estimate of the peak fish that can be produced by the ecosystem when subjected to a transient increase of fishing intensity. Although most of these estimates will individually include uncertainties on the order of a factor of two, arising from uncertainty in catch reconstructions [65], the large number of LMEs and their global distribution should provide sufficient sampling to compensate these errors.

**Fig 2:**
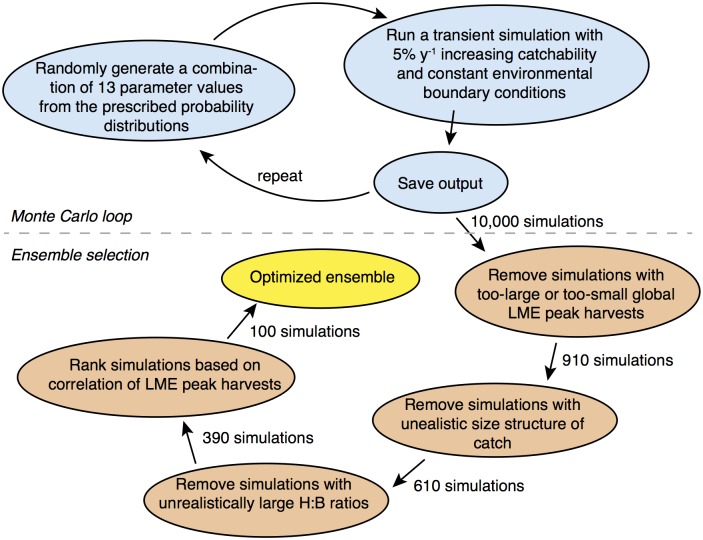
Schematic diagram of the Monte Carlo simulation procedure and selection of ensemble members.

To evaluate the full uncertainty range of model parameters, including interactions between the parameters, we used a Monte Carlo approach [66]. This involved first determining a probability distribution for each parameter of interest, and then randomly generating over 10,000 values for each parameter from each distribution (Fig 2). In order to compare with the observed harvest peaks, ${}_{l}H_{\text{LMEmax}}^{\text{obs}}$, we ran a transient simulation for each parameter combination, gradually increasing catchability over 200 years. In the following, we describe the simulations, the data, and the comparison between them.

#### Transient simulations with increasing catchability

The parameters considered in the Monte Carlo simulations are presented in Table 3, and further detailed in S1 Table. We consider the parameters that have the strongest influence on model biomass, as detailed in the full description of the ecological module [24]. For each parameter, we reviewed the literature for statistically-estimated ranges of values and their probability distributions. When such a statistical range was not available, we used the literature to inform the parameter choices assuming a uniform probability distribution.

**Table 3:** Monte Carlo simulation parameter summary results. MC Distribution is the sampling distribution of each parameter used in the Monte Carlo simulation, where X(*p*_1_, *p*_2_) represents the probability distribution (N for normal, U for uniform), and *p*_1_ and *p*_2_ represent the mean and standard deviation of the parameter, respectively. Opt. refers to the subset of optimized Monte Carlo simulations, N.O. to the subset of non-optimized simulations, SD refers to the standard deviation, and KS *p*-value is the *p*-value of the 2-sample Kolmogorov-Smirnov test applied to the optimized and non-optimized sets.

Parameter	Name	Sampling Distribution	Mean Opt.	Mean N.O.	SD Opt.	SD N.O.	KS *p*-value
*ω*_*a*,*A*_	Growth activation energy	N(0.45, 0.09)	0.41	0.44	0.08	0.09	2.77 × 10^−4^
*ω*_*a*,*λ*_	Mortality activation energy	N(0.45, 0.09)	0.47	0.45	0.08	0.09	0.05
*b*	Allometric scaling exponent	U(0.7, 0.05)	0.65	0.70	0.03	0.05	1.47 × 10^−16^
*A*_0_	Allometric growth constant	N(0.46, 0.5)	4.42	4.47	0.48	0.50	0.18
*α*	Trophic efficiency	U(0.13, 0.04)	0.15	0.13	0.03	0.04	1.04 × 10^−11^
*β*	Predator to prey mass ratio	U(5000, 2500)	5484	4991	2357	2507	0.08
*τ*	Trophic scaling	log(*α*)/log(*β*)	−0.22	−0.25	0.02	0.04	5.27 × 10^−17^
*k*_*E*_	Eppley constant	N(0.0631, 0.009)	0.06	0.06	0.01	0.01	0.34
Π*	Nutrient concentration	N(0.37, 0.1)	0.35	0.37	0.09	0.10	0.06
*ζ*_1_	Mortality constant	N(0.55, 0.57)	0.01	0.55	0.40	0.57	9.48 × 10^−18^
*h*	Allometric mortality scaling	N(0.54, 0.09)	0.49	0.54	0.08	0.09	4.39 × 10^−5^
*s*_*e*_	Egg survival fraction	U(0.025, 0.014)	0.02	0.03	0.01	0.01	0.22
*e*_*m*_Θ_,*k*_	Selectivity position scaling	U(1, 0.288)	0.88	1.00	0.29	0.29	8.89 × 10^−7^
*c*_*σ*_	Selectivity slope	U(18, 3.46)	17.8	18.0	3.46	3.47	0.84

For each parameter set, we first spin up the model with a 100-year simulation at a low and constant level of catchability (1 × 10^−5^ m^2^ W^−1^ s^−1^), which is designed to result in negligible harvests that are consistent with that set of parameters. To span an appropriate range of catchability values, following the first 100 years of constant catchability, we increase catchability by 5% per year for 200 years. This rate of increase ensures that each simulation will produce a peak harvest under all parameter combinations, and is broadly consistent with estimates of technological increase [53].

#### Observational data

Under intense harvest, the harvest to biomass ratio reflects the ability of a standing stock to produce harvestable biomass each year. We obtained harvest and biomass data from the RAM Legacy stock assessment database [63], and used them to calculate harvest to biomass ratios (H:B hereafter). Stock assessments, by their nature, target individual species in particular geographic regions. Because BOATS represents all commercial species, its results can only be compared with stock assessments when they represent a significant fraction of the total ecosystem. Analysis of the RAM Legacy stock assessment database demonstrated that there were a total of 8 LMEs where at least 40% of the of the harvest from the SAUP database were represented: Baltic Sea, Barents Sea, Patagonian Shelf, Benguela Current, North Sea, Okhotsk Sea, Gulf of Mexico, and East Bering Sea. For each of these LMEs, we calculated average harvest and biomass from the years that were common to all of the stocks represented.

We calculate peak harvest using the Sea Around Us Project (SAUP) database [60, 61] aggregated over functional groups at the scale of Large Marine Ecosystems (LMEs). To estimate the peak harvest at each LME, ${}_{l}H_{\text{LMEmax}}^{\text{obs}}$, we take the mean harvest of the 10 years with largest harvest. To compare observed LME peak harvests to those simulated by BOATS, ${}_{i,l}H_{\text{LMEmax}}^{\text{sim}}$ where *i* is an index for the Monte Carlo simulations, we convert both to area-specific LME harvests [67], and ignore a number of high latitude Large Marine Ecosystems (LMEs) due to well-established biases in chlorophyll estimates [68, 69] which carry through to estimates of net primary production. We therefore neglect the following LMEs: Antarctica, Kara Sea, Laptev Sea, East Siberian Sea, Canadian Eastern Arctic—West Greenland, Hudson Bay Complex, Beaufort Sea, Canadian High Arctic—North Greenland, Central Arctic, and Northern Bering—Chukchi Seas. Since the Black Sea is an inland sea ecosystem, and not comparable to the other LMEs, we ignore it as well [67]. The harvest in these 11 LMEs is a negligible fraction (< 1%) of the total harvest over all LMEs, and so ignoring them has a negligible impact on our analysis. S3 Table lists all of the LMEs.

The fish biomass modeled in BOATS includes three groups defined by asymptotic size: small (S), medium (M), and large (L) groups (S4 Table). One of our aims is to compare this simple size-based community structure with the SAUP observations. Although most of the SAUP functional groups clearly denote size, we define an association when this is not the case (eg. large shrimp). For cases where the size group is vague (eg. cephalopods) or there are mixed sizes in a functional group (eg. Small to medium flatfishes) we denote these as other (O). We assume the other (O) fraction of harvest follows the same size distribution as the identified groups, such that

$$\begin{array}{c} \frac{G}{S+M+L}=\frac{G_{a}}{S_{a}+M_{a}+L_{a}}\;\text{and}\;S_{a}+M_{a}+L_{a}=S+M+L+O, \end{array}$$ (12)

where *G* represents a size group (S, M, or L) and the subscript *a* represents the adjusted harvest. This adjustment is usually small since the other (O) fraction of harvest is generally small.

#### Analysis of Monte Carlo suite of simulations

**Fig 3:**
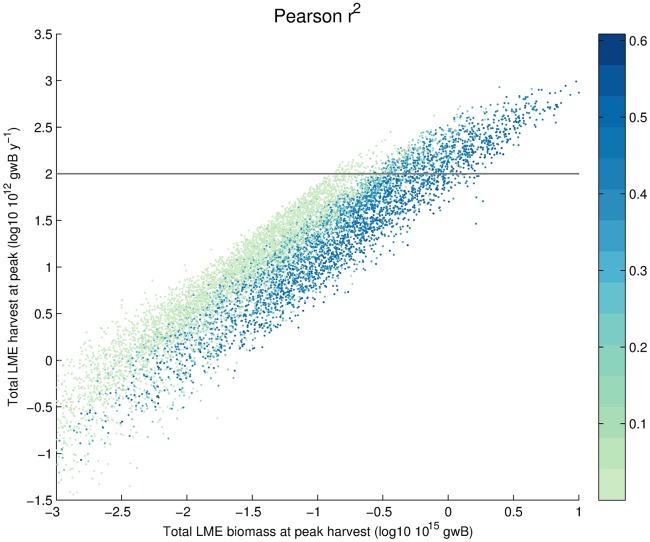
Pearson *r*^2^ of Monte Carlo simulations. Each point represents the global biomass and harvest (${}_{i}H_{\text{Globalmax}}^{\text{sim}}$) integrated over all LMEs for the year at which the global harvest is at a maximum, while the color represents the Pearson *r*^2^ calculated for the modeled area-specific individual LME harvests (${}_{i,l}H_{\text{LMEmax}}^{\text{sim}}$) compared to the peak area-specific individual LME harvest from the SAUP (${}_{l}H_{\text{LMEmax}}^{\text{obs}}$). The grey line represents a reference global harvest of 100 × 10^12^ gwB y^−1^.

**Fig 4:**
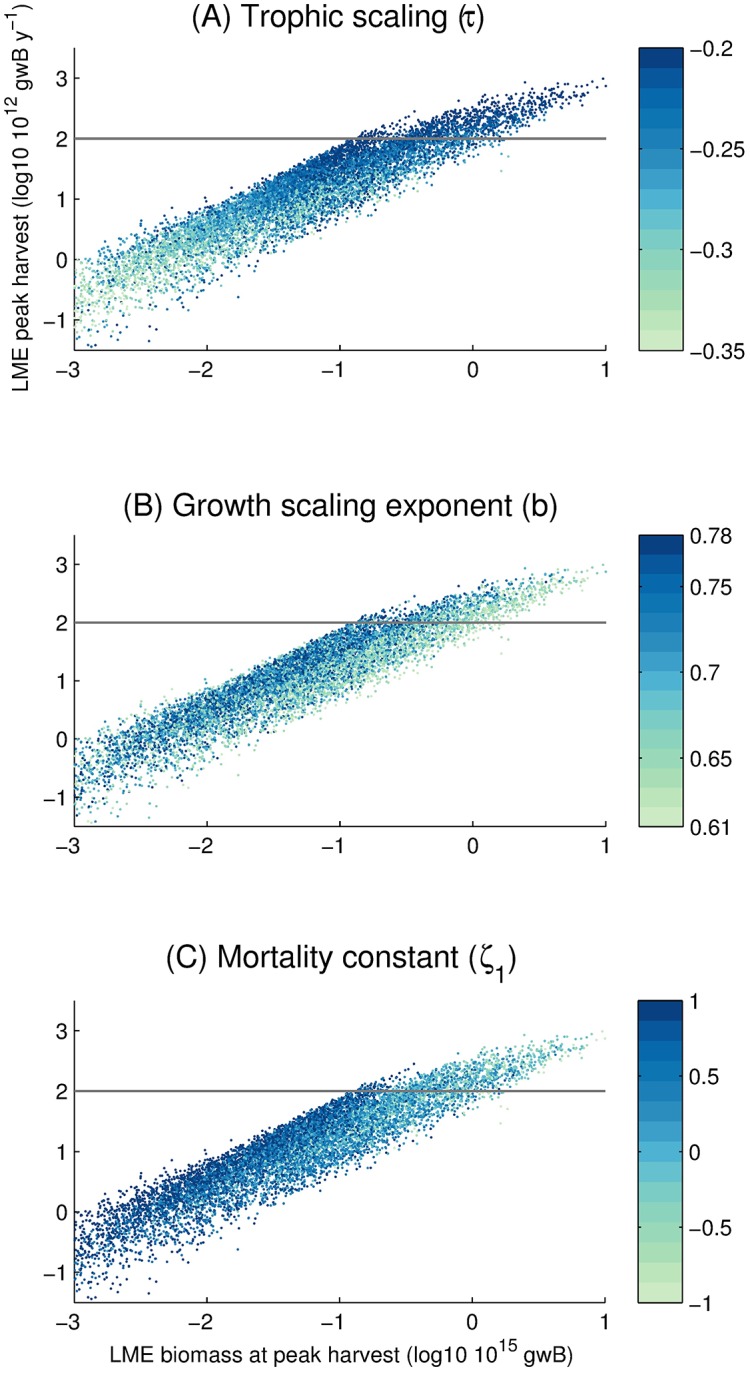
Summary results of Monte Carlo simulations. Horizontal and vertical axes as in Fig 3. Circle color represents (A) Trophic scaling *τ*, (B) Allometric growth scaling constant *b*, or (C) Mortality constant *ζ*_1_. The grey line represents a reference global harvest of 100 × 10^12^ gwB y^−1^.

The Monte Carlo simulations (Table 3) reveal a wide range of biomass and harvest results. Fig 3 displays the maximum global harvest (which we denote as ${}_{i}H_{\text{Globalmax}}^{\text{obs}}$) and biomass integrated over all LMEs. Biomass ranges over 5 orders of magnitude, has a mean and standard deviation of 0.22 and 0.63 Gt wet biomass, respectively, and is strongly positively skewed (that is, there are relatively more randomly-generated parameter combinations that produce low biomass (S4 Fig). The smallest biomass values are mostly attributable to a low trophic scaling (Fig 4A) or a high mortality constant (Fig 4C). Meanwhile, the ${}_{i}H_{\text{Globalmax}}^{\text{sim}}$ values range over more than 4 orders of magnitude (the smallest 3% of the runs were not included in Fig 3, to improve readability). The mean and standard deviation of harvest are 40 and 79 Mt y^−1^, respectively, and as with biomass, the harvest distribution is strongly positively skewed (S4 Fig). It is notable that, despite the 4-order of magnitude range in the ${}_{i}H_{\text{Globalmax}}^{\text{sim}}$ across the full Monte Carlo suite, the mean of the ${}_{i}H_{\text{Globalmax}}^{\text{sim}}$ is less than a factor of 2 different than the reconstructed value of 64 Mt y^−1^ [60, 61], suggesting that our priors were not unreasonable.

We then select 100 optimal parameter combinations, representing approximately 1% of the total, by discarding any that violate acceptable ranges among four types of constraints:

1. Global harvest integrated over all LMEs for the year of maximum harvest, $H_{\text{Globalmax}}^{\text{obs}}$. There are significant uncertainties in the results reported in the SAUP harvest database [70], particularly in the corrections for illegal, unreported and under-reported harvest. We therefore consider a generous estimate of the uncertainty, allowing the ${}_{i}H_{\text{Globalmax}}^{\text{sim}}$ to range between 70 and 150 × 10^12^ gwB y^−1^. We find that 9.1% of the simulations satisfy this constraint, while 84.5% and 6.4% have too small and too large a peak harvest, respectively.
2. Size structure of catch. Founded on analysis of the SAUP database, we select simulations where the ${}_{i}H_{\text{Globalmax}}^{\text{sim}}$ for the medium size group is at least 30% that of the small group harvest, and those where the large harvest is at least 10% but less than 80% of the small harvest. This guarantees that the modeled harvest is not unrealistically dominated by a single group. We find that 29.1% of the simulations satisfy the group constraints, which, when taken together with the total harvest constraint, leaves us with 6.1% of the total simulations.
3. The harvest to biomass (H:B) ratio of well-assessed ecosystems. In our analysis of the RAM legacy stock assessment database, we found a wide range of H:B among the 8 analyzed LMEs, but in all cases, it was less than 0.4 y^−1^. We apply this constraint by calculating the modeled H:B at peak harvest (${}_{i,l}H_{\text{LMEmax}}^{\text{sim}}$) at each of the 8 LMEs. 74.2% of the Monte Carlo simulations satisfy this H:B constraint when applied to the 8 LMEs. Taken together with the prior two constraints, we arrive at 3.9% of the simulations. Although there is great potential to use improved stock assessment data in the future, for the present work we only apply the upper limit on the feasible H:B of these 8 LMEs.
4. The relative productivity of LMEs. We rank the remaining simulations by calculating the correlation between the modeled peak harvest of each LME (${}_{i,l}H_{\text{LMEmax}}^{\text{sim}}$) and the reconstructed peak LME harvest from the SAUP (${}_{l}H_{\text{LMEmax}}^{\text{obs}}$). Because the LMEs span a wide range of NPP and temperature, analyzing the simulated distribution of harvest among the LMEs provides a powerful means by which to evaluate model sensitivity to these two critical input variables. We consider the Pearson product-moment correlation coefficient *r* (plotted as *r*^2^), of the harvest per unit area in each LME, but also the log10 transformed harvest per unit area [67] (not shown), as well as Spearman’s rank correlation *r*_*s*_. We find a wide range of correlation values from among the members of the Monte Carlo suite, but there are clear patterns of improving correlation with increasing biomass within the range of acceptable harvest which is consistent over the three correlation techniques considered (Fig 3). From among the simulations with acceptable total harvest, the *r*^2^ ranges from close to zero to 0.59, whereas Spearman’s *r*_*s*_ ranges from −0.42 to 0.76. We select the best-correlated 1% of the total simulations, corresponding to an *r*^2^ larger than 0.45.

**Fig 5:**
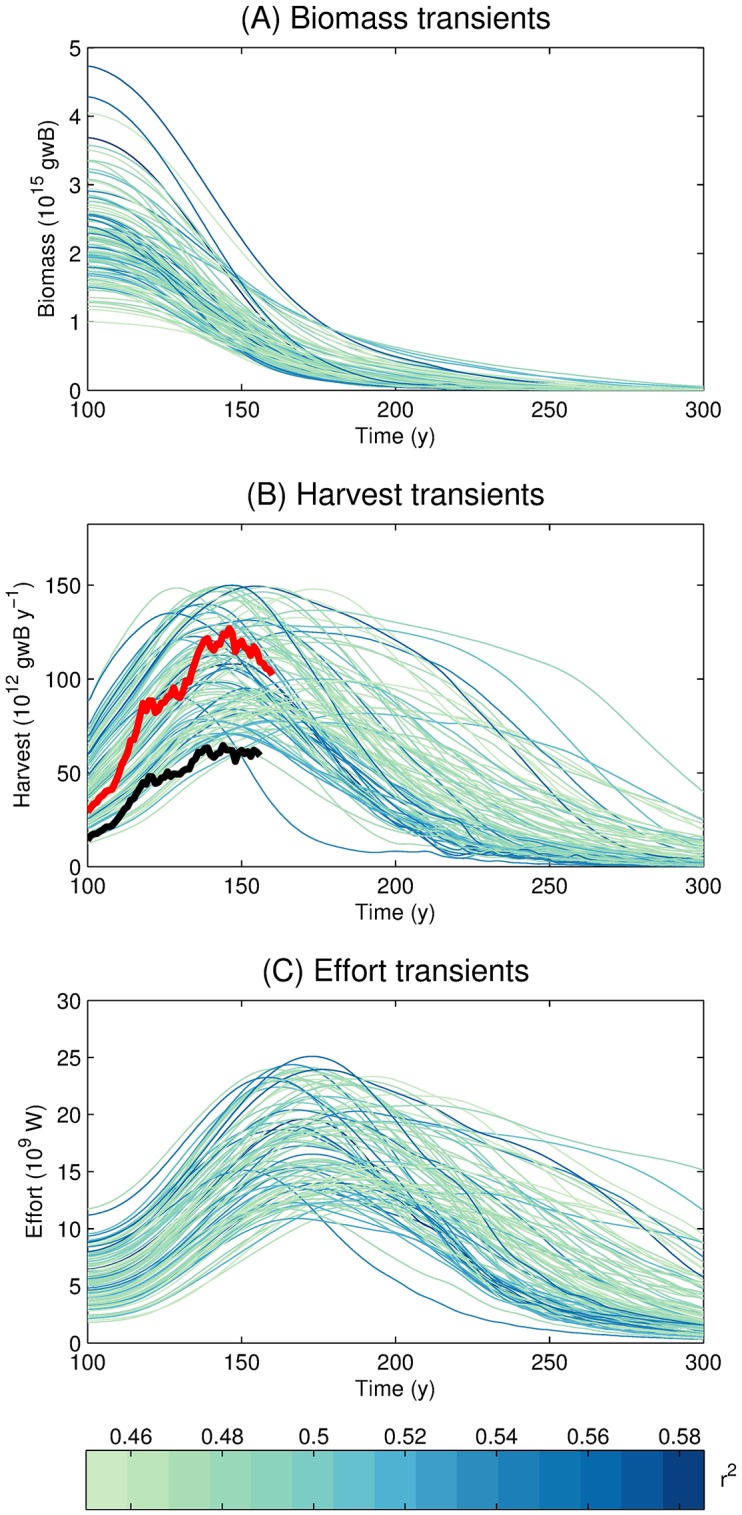
Total (A) biomass, (B) harvest, and (C) effort over all LMEs for the optimized Monte Carlo transient simulations. Line colors represent, for each optimized ensemble member *i*, the Pearson *r*^2^ of the area-specific BOATS harvest at each LME *l* (${}_{i,l}H_{\text{LMEmax}}^{\text{sim}}$) and the peak harvest of each LME based on the SAUP (${}_{l}H_{\text{LMEmax}}^{\text{obs}}$). Black [60, 61] and red [71] lines in (B) correspond to two versions of the reconstructed SAUP harvest, assuming that the year 1950 of the SAUP harvest corresponds to the time in our transient simulations when catchability begins to increase.

Table 3 summarizes the results of the analysis, and presents the mean of the optimized (column Mean Opt.) and non-optimized (column Mean N.O.) parameter values, as well as their standard deviations (columns SD Opt. and SD N.O.). Fig 5 shows the transient time series of total LME fish biomass, harvest, and effort for the optimized set. Total LME harvest steadily increases until it reaches a peak, beyond which a sufficient portion of the global ocean becomes recruitment-limited and harvest begins to fall. All simulations show a monotonic decrease of biomass as catchability increases, since the selectable biomass is progressively reduced towards a limit, the critical biomass, that is discussed in the next section. This occurs in an increasing number of grid cells as greater catchability allows fishing to become profitable. Effort follows the same general pattern as harvest, but consistently lags it. The timescale of biomass and effort change is set mostly by the timescale of technology change, yet effort also responds with the timescale of adjustment to revenue changes (the fleet dynamics parameter *κ*_*e*_) which delays the change of effort relative to biomass and harvest changes (Eq (13)). We overlay the optimized ensemble of simulated harvests in Fig 5B with two versions of the reconstructed SAUP harvest (black line [60, 61], red line [71]), assuming that year 1950 of the SAUP harvest corresponds to year 100 of the 300-year transient simulation (when catchability begins to increase). Both reconstructions show the same general trend as the simulated transient simulations over the historical period, and the red line (which attempts to correct for illegal and unreported catch) is close to the median of the simulations. Although the highest simulated values are possibly over-estimates of the global potential fish production, it is useful to include these variants in the ensemble given that their parameter combinations may represent aspects of the ecosystems realistically, even though they allow too much harvest.

## Results and Discussion

### The critical fish biomass

In considering the fundamental dynamics that arise from the BOATS formulation, we arrive at a useful observation regarding the equilibrium solution of Eq (6) for the evolution of effort. We begin with the equations for revenue and cost, Eqs (9) and (10), respectively, and apply them to Eq (6) to find that

$$\begin{array}{c} \frac{d}{\text{dt}}E_{k}=\kappa_{e}(q_{k}\int_{m_{0}}^{m_{\infty ,k}}p_{k}\sigma_{k}f_{k}dm-c_{k}). \end{array}$$ (13)

Further assuming that price does not depend on mass, we can write the equilibrium solution of Eq (13) as

$$\begin{array}{c} \int_{m_{0}}^{m_{\infty ,k}}\sigma_{k}f_{k}dm=\frac{c_{k}}{p_{k}q_{k}}=F_{k}^{\text{crit}}. \end{array}$$ (14)

This states that the selectable biomass (the quantity on the left hand side) of group *k* is, at steady state, determined solely by the ratio of economic parameters, *c*_*k*_/*p*_*k*_*q*_*k*_. This ratio can also be interpreted as a critical threshold, which we refer to as $F_{k}^{\text{crit}}$, which determines the biomass density that is required at any given grid point in order for harvest to be profitable. If the equilibrium biomass ignoring harvest is less than $F_{k}^{\text{crit}}$, then harvest cannot be profitable and there will be no harvest at equilibrium. In this case, the biomass will be controlled by primary production and temperature, by means of Eq (1). Alternatively, if the selectable group biomass exceeds $F_{k}^{\text{crit}}$, harvest will occur, driving the selectable biomass to asymptotically approach $F_{k}^{\text{crit}}$, while the unselectable portion of the biomass will continue to be driven by primary production and temperature.

The exclusive dependence of $F_{k}^{\text{crit}}$ on economic parameters is an important consequence of the open access assumption used in the present version of the BOATS model. Although it would not be expected to hold true in the real world, given for example the movement of fish, multi-species fisheries, and the lack of steady-state, it is a useful simplifying concept for explaining the first-order tendencies of fish biomass in the absence of effective management.

### General characteristics of the optimized bio-economic model

In order to illustrate the basic characteristics of the coupled model, we use the parameter combination that gives the highest correlation (*r*^2^) between modeled and observed harvests at the scale of LMEs. While we initially focus on results with this single set of model parameters for clarity, it is preferable to use an ensemble of optimal models, in order to better span the uncertainty.

**Fig 6:**
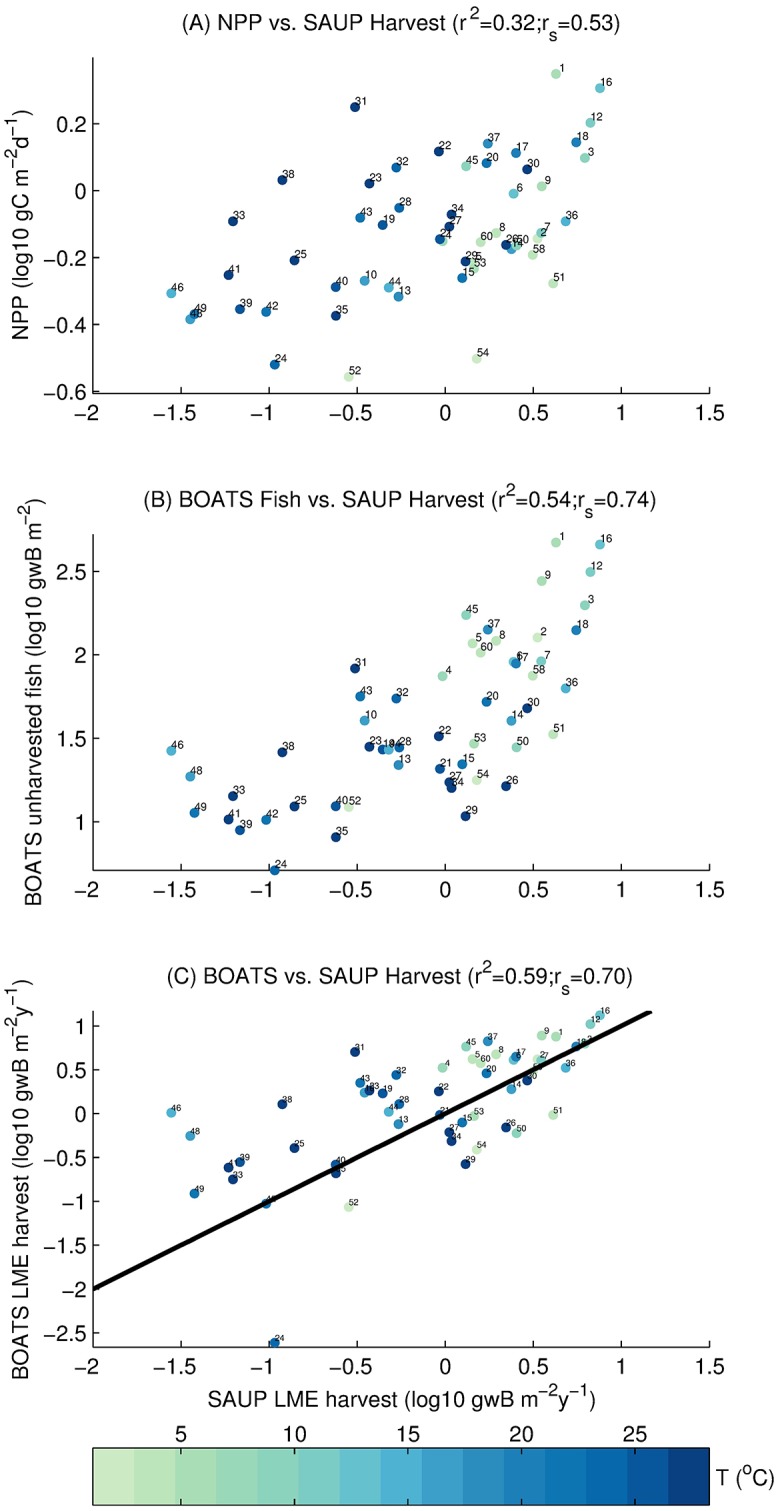
SAUP reconstructed harvest as described by primary production, modelled unharvested biomass, and modelled harvest at the LME scale. Correlations are for the model variant from the Monte Carlo simulation that best represents the SAUP database. The circle color represents the average temperature of the LME, whereas the number represents the LME number (S3 Table). All quantities are plotted in log10 space to improve readability. *r*^2^ is the squared Pearson product-moment correlation coefficient, and *r*_*s*_ is Spearman’s rank correlation. All 6 correlations have a *p*-value<0.01. (A) NPP vs. SAUP harvest. (B) Modelled unharvested biomass vs. SAUP harvest. (C) Modelled harvest at peak harvest state vs. SAUP harvest. The black line is the identity line (1:1 line).

The predictive ability of this model variant can be assessed by comparing the correlation of the SAUP harvest with the primary production used to force the model (Fig 6A), the simulated unharvested LME biomass by LME (Fig 6B), and the modeled harvest (Fig 6C). We find that NPP alone explains 32% of the variability in the SAUP harvest, similar to prior works [72], while unharvested model biomass explains 54%, and modeled harvest explains 59%. All correlations have a *p*-value<0.01. Thus, the inclusion of the interactive economics provides an improvement to the prediction of harvest, even though it did not introduce additional parameters into the optimization procedure.

**Fig 7:**
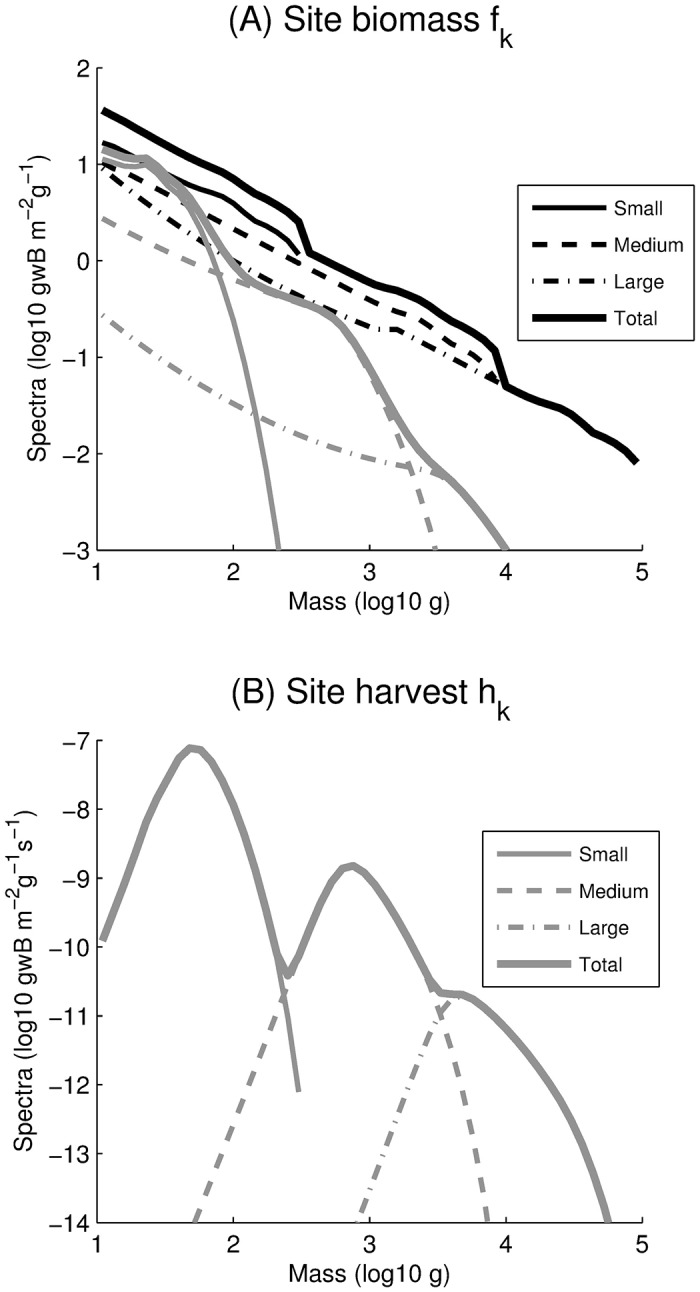
Steady state biomass (A) and harvest (B) size spectra. Black solid, dashed, and dot-dashed curves represent unharvested small, medium, and large groups, respectively, whereas grey curves represent harvested spectra. Thick curves represent total biomass and harvest spectra. Curves are equilibrium solutions of simulations for a site in the East Bering Sea LME (64°N, 165°W) forced with annual average net primary production and temperature using a timestep of 15 days.

Fig 7 illustrates some aspects of the model by focusing on a single model grid point in the East Bering Sea LME (64°N, 165°W). A detailed description of the ecological model at this site is provided in [24], and showed that the slope of modeled biomass spectra is consistent with observations for a wide range of temperature and NPP values. Here, harvest alters the slope and intercept of biomass spectra (Fig 7A), truncating the large ends of the spectra, consistent with other modeling studies [73]. The truncation occurs because larger fish must grow from smaller fish, and so larger fish are affected both by direct harvesting and the reduction in growth from smaller fish. Moreover, the loss of large, spawning individuals results in a reduced production of eggs and so a reduction in recruitment (Eq (5)) and juvenile biomass. Harvests peak near the threshold size *m*_Θ_ (Fig 7B), and decline both at smaller sizes (because of sharply decreasing selectivities), and at larger sizes (because of rapidly decreasing biomasses and growth rates).

It is worth noting that, at low harvest and high biomass, the input energy from the transfer of primary production to fish *ξ*_*P*_ (Eq (2)) is partitioned across a larger number of individual, and is generally the limiting factor for growth. On the other hand, at high harvest and low biomass, the same energy from primary production is partitioned to a smaller number of individuals, increasing their potential growth rates, to the point at which growth becomes limited by the maximum physiological rate, expressed by *ξ*_*VB*_. Thus, all else being equal, increasing harvest and decreasing biomass determines a transition from productivity-limited to physiologically-limited growth in the model.

**Fig 8:**
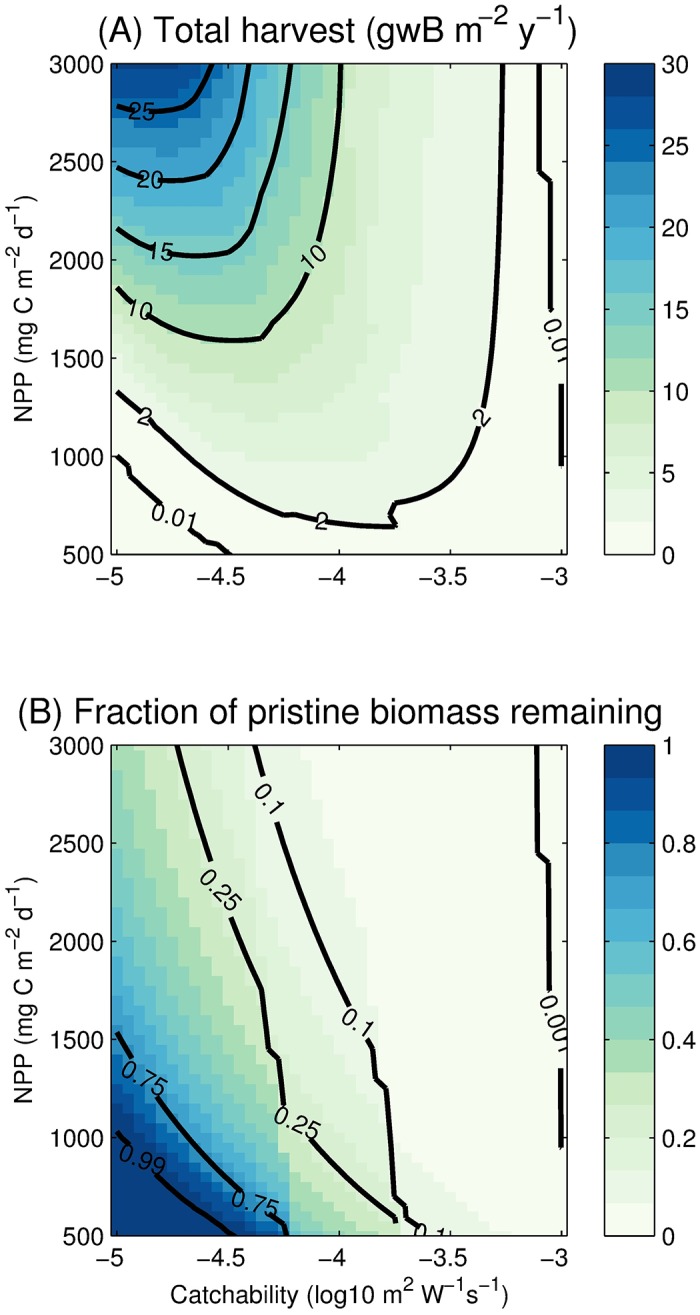
Model sensitivity to NPP and catchability *q*. (A) Total harvest and (B) Fraction of pristine biomass remaining as functions of NPP and *q*.

Fig 8 illustrates the impact of NPP (ranging from 500 to 3000 mg C m^−2^ d^−1^) and catchability (ranging from 10^−5^ to 10^−3^ m^2^ W^−1^ s^−1^) on biomass and harvest. Each harvest (Fig 8A) and fraction of prisitine biomass remaining (Fig 8B) value is the equilibrium result of a 1000-year simulation using a 15-day timestep and constant forcing of annually-averaged NPP and temperature. For low catchability and low NPP (and hence low unharvested biomass), harvest is zero (0.01 gwB m^−2^ y^−1^ contour in Fig 8A) because the fishery is not profitable (Eq (14)). With increasing catchability or increasing NPP, we eventually surpass the $F_{k}^{\text{crit}}$ limit for a profitable fishery, and arrive at positive harvest. For a given level of NPP, increasing catchability initially results in rising harvest, until the peak is reached, after which further increases of catchability deplete the ability of the population to produce biomass due to stock-limitation of recruitment [74]. As a result, there is a peak equilibrium harvest for every value of NPP. For high catchability, both harvest and the fraction of pristine biomass approach zero (0.01 gwB m^−2^ y^−1^ and 0.001 contours at the right of Fig 8A and 8B, respectively).

### Insights on uncertain parameters

We use the two-sample Kolmogorov-Smirnov test (Table 3, column KS *p*-value) to compare the distributions of the optimized and non-optimized parameters to determine if they are generated from the same distribution ([75], p. 428-441). For 7 parameters, we find no difference in the distributions that generate the optimized and non-optimized simulations. However, for the remaining 6 parameters, there is a clear statistical difference (that is, small *p*-values). Prominent differences are in the mortality parameters *ζ*_1_ (Fig 4C) and *h*, that have significantly lower means and standard deviations. The difference between the optimized and non-optimized mortality rate *ζ*_1_ is particularly strong. This indicates that, in our model, only the lower range of possible mortality rates [26] can reproduce the present-day global peak harvest distributions. The optimized mean value of the allometric scaling parameter, *b*, which we assume follows a uniform distribution ranging from 0.61 to 0.78, is 0.65, that is, indistinguishable from the 2/3 value that is widely used in the literature [76], but somewhat smaller than the 0.75–0.79 value of other studies [25, 77]. We also find a preferred trophic efficiency of 0.15, statistically higher than that of the sampling distribution, and encouragingly close to the value of 0.15 that is widely applied. The activation energy of metabolism for mortality, *ω*_*a*,*λ*_, is not statistically different from the sampling distribution, but that for growth *ω*_*a*,*A*_ is, with a statistically significant lower mean. This lends support to the idea that growth and mortality rates are not subject to the same temperature dependence [78], and suggests that mortality may be more sensitive to temperature variations than growth.

**Fig 9:**
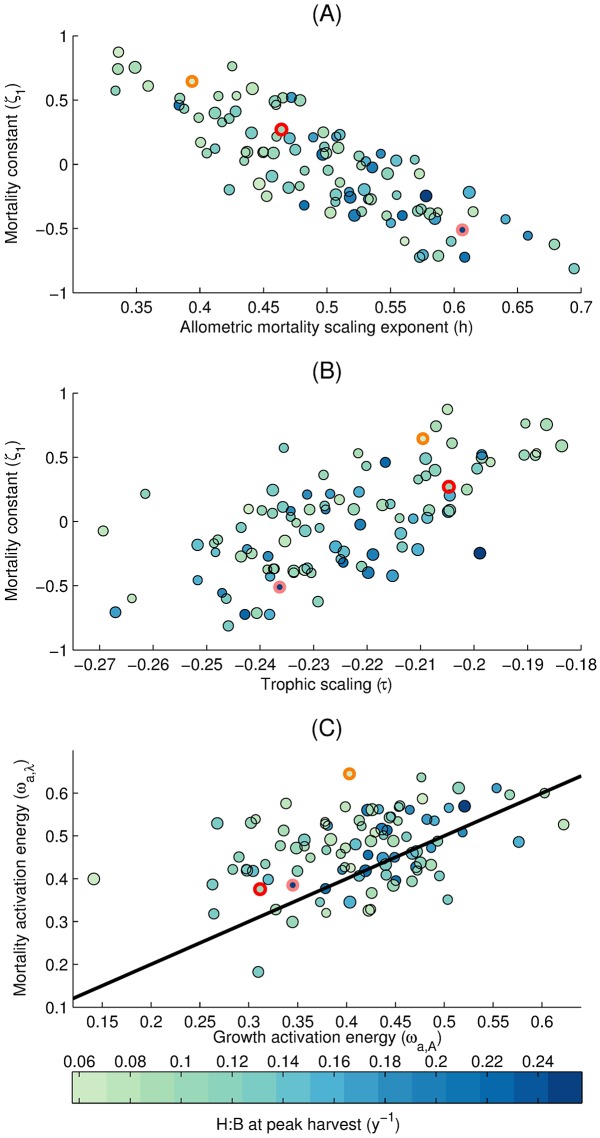
Harvest to biomass ratio (H:B) at peak harvest in terms of three sets of parameter combinations. Circles are members of the optimized set of model parameters. Circle color represents the H:B (*y*^−1^) calculated as the total harvest (${}_{i}H_{\text{Globalmax}}^{\text{sim}}$) over the total biomass at the year of global peak harvest integrated over the LMEs, while the circle size is proportional to the peak harvest. Horizontal and vertical axes correspond to parameter values from the Monte Carlo suite. Thick orange, red, and peach circles correspond to the low, medium, and high H:B members that are used in Fig 10. The black line in (C) is the identity line (1:1 line).

The ensemble optimization reveals that acceptable parameter combinations have significant correlations between some model parameters. Some of these correlations, including the two strongest ones, are shown in Fig 9. The mortality constant, *ζ*_1_, and the allometric scaling of mortality, *h*, are negatively correlated in the ensemble (Pearson correlation coefficient *r* = −0.82, *p*-value<0.01) and compensate each other so that a high mortality constant is balanced by a weak size-dependence (low *h*) of mortality (Fig 9A). We also find a positive correlation (*r* = 0.68, *p*-value<0.01) between the mortality constant and the trophic scaling (Fig 9B) suggesting that higher mortalities can be balanced by increased energy transfer to large size classes. Finally, we highlight a positive correlation (*r* = 0.41, *p*-value<0.01) between the activation energies of growth and mortality (Fig 9C), which control the temperature dependence of these rates. This covariation indicates that, in order to maintain harvests in agreement with observations, temperature-driven increases in mortality need to be balanced by concurrent increases in growth rates. As noted above, the temperature dependence of mortality generally exceeds the temperature dependence of growth, with 80% of the optimized ensemble lying above the 1:1 line.

Fig 9 also highlights the impact of several fundamental model parameters on simulated H:B. Low mortality constants, high allometric growth exponents, and high growth rate constants tend to occur with high H:B (*r* between *ζ*_1_, *b*, and *A*_0_, respectively, with H:B are −0.49, 0.65, and 0.30, *p*-value<0.01). Across the 105 members of the optimized Monte Carlo set, the H:B calculated for the global peak harvest (calculated by taking the ratio of total harvest over the LMEs, ${}_{i}H_{\text{Globalmax}}^{\text{sim}}$, to the total biomass over the LMEs) varies by approximately a factor of 5, indicating that between 5 and 25% of the standing stock of biomass is harvested every year at the peak. This range indicates that, within the limits allowed by the observational constraints, similar harvest can be sustained by substantially different levels of biomass.

Motivated by the range in model H:B, we select three members from the optimized ensemble that represent low, medium, and high values of this quantity, to analyze the spatial patterns of harvest and biomass at the catchabilities corresponding to the peak harvest over the LMEs. The low and high variants are selected based on their H:B and their similar global peak harvest (calculated to be 214 and 193 × 10^12^ gwB y^−1^, respectively, when including the LMEs as well as the high seas), whereas the medium member is the parameter set that provides the best correlation with SAUP data (described above). When integrated over the LMEs, the total harvest for the low, medium, and high variants are 122, 102, and 86 × 10^12^ gwB y^−1^, respectively, corresponding to total biomasses of 1.6, 1.5, and 0.2 × 10^15^ gwB.

**Fig 10:**
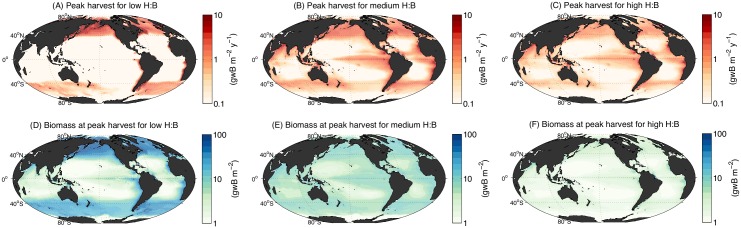
Two-dimensional global maps of harvest and biomass for three model variants with varying global harvest to biomass ratio (H:B). Maps show the annual average of the year of peak harvest. Harvest for low (A), medium (B), and high (C) H:B. Biomass for low (D), medium (E), and high (F) H:B.

Maps (Fig 10), and cumulative harvest distributions (S5 Fig), of the peak harvest and biomass state demonstrate fundamental differences in spatial patterns between the members. Harvest in the low H:B member is dominated by high latitude, high biomass regions with slow fish production, whereas harvest in the high H:B member is mostly in low latitude, low biomass regions with rapid fish production. The spatial concentration of harvest also differs dramatically, with over 60% of harvest taking place at locations with high harvest rates (above 3 gwB m^−2^ y^−1^) in the low H:B member, whereas harvest is much more widely distributed in the high H:B member, with only about 25% of harvest taking place at location with high harvest rates. Because of important differences in the model sensitivity to temperature (Fig 9C), contrasting projections of climate change impacts in low versus high latitude ecosystems [79], as well as potentially different resilience of low and high biomass conditions to overfishing, it will be important to further constrain the uncertainty in H:B.

The significant variability of H:B among the 100-member ensemble arises in part from the generous ranges of observational uncertainty applied in the parameter optimization. Expanding and strengthening the observational constraints, for example from stock assessments aggregated at the LME scale, will further narrow the uncertainty in model parameters, and reduce the ensemble range of H:B. Future work should therefore strive to find stronger constraints on harvest and biomass, such as by focusing on a subset of the best-studied LMEs.

## Conclusion

The BOATS model represents the first-order features of global fish biomass and economic harvest using fundamental concepts of macroecology, life-history, and resource economics in a unified spatially-resolved framework. The economic module of BOATS is directly coupled to the biophysical model by means of the fishing effort, which responds dynamically to net profits at the grid scale. Given the complex nature of real-world ecosystems and of the social and economic processes that determine fishing effort and harvest, the ability of BOATS to explain a significant amount of the global spatial variability in harvest is promising, and makes it suitable for exploratory global studies of fisheries dynamics that include both human and environmental forcings.

A distinctive feature of this work is the use of a Monte Carlo approach to parse the uncertainty in the model parameters and to determine which parameter sets generate the best representations of present-day harvest from the Sea Around Us Project database. We find that although most parameter combinations among the probability distributions of our priors are unable to produce the observed fish harvest, the central values of the distributions are associated with a global harvest that is reasonably close to observations. By discarding unacceptable parameter combinations using a series of catch and biomass criteria, we arrive at an optimal ensemble of about 100 parameter combinations that perform well, while still retaining a substantial degree of parameter uncertainty. These optimal parameter combinations tend to include rates of natural mortality among the lower range of the sampling distribution, a result that deserves further attention given that natural mortality rates are difficult to constrain from observations. We also find that the temperature dependence of mortality is most often greater than the temperature dependence of fish growth, which has important implications in a warming ocean. Realistic global harvests can be achieved by parameter combinations that result in different biomass distributions, and range from solutions dominated by slowly-growing abundant biomass in high latitudes and coastal regions, to solutions dominated by fast-growing low biomasses in low-latitudes and open-ocean waters. These remaining uncertainties allow a potential range of responses of the global fishery to human and climate-driven perturbations, which could be further narrowed down by considering improved biomass-based observational constraints.

## Supporting Information

S1 Fig Annual average net primary production (mg C m^−2^ d^−1^) forcing applied in BOATS. (PDF)

S2 Fig Annual average 75-meter average temperature (°C) forcing applied in BOATS. (PDF)

S3 Fig Harvest selectivity *σ*_*k*_ by group. (PDF)

S4 Fig Histograms of LME-integrated biomass and harvest from the Monte Carlo suite. (PDF)

S5 Fig Cumulative fraction of global harvest integrated over all grid cells with a harvest rate less than a threshold harvest rate for low, medium, and high harvest to biomass ratio (H:B) model variants. (PDF)

S1 Table Ecological model parameters. (PDF)

S2 Table Gear types in terms of selectivity functional form (S = sigmoidal, G = gaussian, O = other) and percentage of global harvest (Table 3 of [55]). (PDF)

S3 Table Large Marine Ecosystem numbers and names. (PDF)

S4 Table Partitioning of SAUP functional groups into small (S), medium (M), large (L), and other (O) size groups. (PDF)

## References

[1] Watson RA, Cheung WWL, Anticamara JA, Sumaila RU, Zeller D, Pauly D. Global marine yield halved as fishing intensity redoubles. Fish and Fisheries. 2013;14(4):493–503. 10.1111/j.1467-2979.2012.00483.x

[2] Myers RA, Worm B. Rapid worldwide depletion of predatory fish communities. Nature. 2003;423(6937):280–283. 10.1038/nature01610 12748640

[3] McCauley DJ, Pinsky ML, Palumbi SR, Estes JA, Joyce FH, Warner RR. Marine defaunation: Animal loss in the global ocean. Science. 2015;347(6219):1255641 10.1126/science.1255641 25593191

[4] Mullon C, Freon P, Cury P. The dynamics of collapse in world fisheries. Fish and Fisheries. 2005;6(2):111–120. 10.1111/j.1467-2979.2005.00181.x

[5] Branch TA, Jensen OP, Ricard D, YE Y, Hilborn R. Contrasting global trends in marine fishery status obtained from catches and from stock assessments. Conservation Biology. 2011; 10.1111/j.1523-1739.2011.01687.x 21535149

[6] Doney SC, Ruckelshaus M, Emmett Duffy J, Barry JP, Chan F, English CA, et al Climate Change Impacts on Marine Ecosystems. Annual Review of Marine Science. 2012;4(1):11–37. 10.1146/annurev-marine-041911-111611 22457967

[7] Pinsky ML, Worm B, Fogarty MJ, Sarmiento JL, Levin SA. Marine Taxa Track Local Climate Velocities. Science. 2013;341(6151):1239–1242. 10.1126/science.1239352 24031017

[8] Poloczanska ES, Brown CJ, Sydeman WJ, Kiessling W, Schoeman DS, Moore PJ, et al Global imprint of climate change on marine life. Nature Climate Change. 2013;3(10):919–925. 10.1038/nclimate1958

[9] Brander K. Impacts of climate change on fisheries. Journal of Marine Systems. 2010;79(3-4):389–402. 10.1016/j.jmarsys.2008.12.015

[10] Sumaila UR, Cheung WWL, Lam VWY, Pauly D, Herrick S. Climate change impacts on the biophysics and economics of world fisheries. Nature Climate Change. 2011;1(9):449–456. 10.1038/nclimate1301

[11] Squires D, Vestergaard N. Technical Change and The Commons. Review of Economics and Statistics. 2013;95(5):1769–1787. 10.1162/REST_a_00346

[12] Mullon C, Guillotreau P, Galbraith ED, Fortilus J, Chaboud C, Bopp L, et al. Exploring future scenarios for the global supply chain of tuna. Deep Sea Research Part II: Topical Studies in Oceanography. 2016; p. –.

[13] Fulton EA. Approaches to end-to-end ecosystem models. Journal of Marine Systems. 2010;81:171–183. 10.1016/j.jmarsys.2009.12.012

[14] Griffith GP, Fulton EA. New approaches to simulating the complex interaction effects of multiple human impacts on the marine environment. ICES Journal of Marine Science. 2014;71(4):764–774. 10.1093/icesjms/fst196

[15] Kearney KA, Stock C, Aydin K, Sarmiento JL. Coupling planktonic ecosystem and fisheries food web models for a pelagic ecosystem: Description and validation for the subarctic Pacific. Ecological Modelling. 2012;237-238:43–62. 10.1016/j.ecolmodel.2012.04.006

[16] Jørgensen C, Enberg K, Dunlop ES, Arlinghaus R, Boukal DS, Brander K, et al Ecology: Managing Evolving Fish Stocks. Science. 2007;318(5854):1247–1248. 10.1126/science.1148089 18033868

[17] Maury O, Faugeras B, Shin YJ, Poggiale JC, Ben Ari T, Marsac F. Modeling environmental effects on the size-structured energy flow through marine ecosystems. Part 1: The model. Progress in Oceanography. 2007;74(4):479–499. 10.1016/j.pocean.2007.05.002

[18] Jennings S, Mélin F, Blanchard JL, Forster RM, Dulvy NK, Wilson RW. Global-scale predictions of community and ecosystem properties from simple ecological theory. Proceedings of the Royal Society B: Biological Sciences. 2008;275(1641):1375–1383. 10.1098/rspb.2008.0192 18348964 PMC2602712

[19] Cheung WWL, Lam VWY, Sarmiento JL, Kearney K, Watson R, Zeller D, et al Large-scale redistribution of maximum fisheries catch potential in the global ocean under climate change. Global Change Biology. 2010;16(1):24–35. 10.1111/j.1365-2486.2009.01995.x

[20] Blanchard JL, Jennings S, Holmes R, Harle J, Merino G, Allen JI, et al Potential consequences of climate change for primary production and fish production in large marine ecosystems. Philosophical Transactions of the Royal Society B: Biological Sciences. 2012;367(1605):2979–2989. 10.1098/rstb.2012.0231 23007086 PMC3479740

[21] Watson JR, Stock CA, Sarmiento JL. Exploring the role of movement in determining the global distribution of marine biomass using a coupled hydrodynamic—Size-based ecosystem model. Progress in Oceanography. 2014;

[22] Jennings S, Collingridge K. Predicting Consumer Biomass, Size-Structure, Production, Catch Potential, Responses to Fishing and Associated Uncertainties in the World’s Marine Ecosystems. PLoS ONE. 2015;10(7):1–28. 10.1371/journal.pone.0133794 26226590 PMC4520681

[23] Christensen V, Coll M, Buszowski J, Cheung WWL, Frölicher T, Steenbeek J, et al The global ocean is an ecosystem: simulating marine life and fisheries. Global Ecology and Biogeography. 2015;24(5):507–517. 10.1111/geb.12281

[24] Carozza DA, Bianchi D, Galbraith ED. The ecological module of BOATS-1.0: a bioenergetically constrained model of marine upper trophic levels suitable for studies of fisheries and ocean biogeochemistry. Geoscientific Model Development. 2016; p. 1545–1565.

[25] Brown JH, Gillooly JF, Allen AP, Savage VM, West GB. Toward a metabolic theory of ecology. Ecology. 2004;85(7):1771–1789. 10.1890/03-9000

[26] Gislason H, Daan N, Rice JC, Pope JG. Size, growth, temperature and the natural mortality of marine fish. Fish and Fisheries. 2010;11(2):149–158. 10.1111/j.1467-2979.2009.00350.x

[27] Charnov EL, Gislason H, Pope JG. Evolutionary assembly rules for fish life histories. Fish and Fisheries. 2012;14(2):213–224. 10.1111/j.1467-2979.2012.00467.x

[28] Andersen KH, Beyer JE. Size structure, not metabolic scaling rules, determines fisheries reference points. Fish and Fisheries. 2013;

[29] Lehodey P, Senina I, Murtugudde R. A spatial ecosystem and populations dynamics model (SEAPODYM) – Modeling of tuna and tuna-like populations. Progress in Oceanography. 2008;78(4):304–318. 10.1016/j.pocean.2008.06.004

[30] McKendrick AG. Applications of mathematics to medical problems. Proceedings of the Edinburgh Mathematical Society. 1926;3:98–130. 10.1017/S0013091500034428

[31] von Foerster H. Some remarks on changing populations In: Stohlman F Jr, editor. The Kinetics of Cellular Proliferation. New York: Grune and Stratton; 1959.

[32] Ernest SKM, Enquist BJ, Brown JH, Charnov EL, Gillooly JF, Savage VM, et al Thermodynamic and metabolic effects on the scaling of production and population energy use. Ecology Letters. 2003;6(11):990–995. 10.1046/j.1461-0248.2003.00526.x

[33] von Bertalanffy L. Problems of Organic Growth. Nature. 1949;163(4135):156–158. 10.1038/163156a0 18107581

[34] Hartvig M, Andersen KH, Beyer JE. Food web framework for size-structured populations. Journal of theoretical Biology. 2011;272(1):113–122. 10.1016/j.jtbi.2010.12.006 21146543

[35] Rothschild BJ. An exposition on the definition of fishing effort. Fishery Bulletin. 1972;70(3):671–679.

[36] Anticamara JA, Watson R, Gelchu A, Pauly D. Global fishing effort (1950–2010): Trends, gaps, and implications. Fisheries Research. 2011;107(1-3):131–136. 10.1016/j.fishres.2010.10.016

[37] Smith VL. Economics of production from natural resources. The American Economic Review. 1968;58(3):409–431.

[38] Branch TA, Hilborn R, Haynie AC, Fay G, Flynn L, Griffiths J, et al Fleet dynamics and fishermen behavior: lessons for fisheries managers. Canadian Journal of Fisheries and Aquatic Sciences. 2006;63(7):1647–1668. 10.1139/f06-072

[39] Gordon H Scott. The Economic Theory of a Common-Property Resource: The Fishery. Journal of Political Economy. 1954;62(2):124–142. 10.1086/257497

[40] Schaefer MB. Some aspects of the dynamics of populations important to the management of the commercial marine fisheries. Bulletin of the Inter-American Tropical Tuna Commission. 1954;1(2):27–56.

[41] Clark CW. Mathematical Bioeconomics: The Optimal Management of Renewable Resources, Second Edition New York, NY, USA: John Wiley & Sons, Inc.; 1990.

[42] Hardin G. The tragedy of the commons. The population problem has no technical solution; it requires a fundamental extension in morality. Science. 1968;162(859):1243 5699198

[43] Dietz T, Ostrom E, Stern PC. The Struggle to Govern the Commons. Science. 2003;302(5652):1907–1912. 10.1126/science.1091015 14671286

[44] Wilen JE. Renewable Resource Economists and Policy: What Differences Have We Made? Journal of Environmental Economics and Management. 2000;39(3):306–327. 10.1006/jeem.1999.1110

[45] Caddy JF, Cochrane KL. A review of fisheries management past and present and some future perspectives for the third millennium. Ocean & coastal management. 2001;44(9):653–682. 10.1016/S0964-5691(01)00074-6

[46] Costello C, Lynham J, Lester SE, Gaines SD. Economic Incentives and Global Fisheries Sustainability. Annual Review of Resource Economics. 2010;2(1):299–318. 10.1146/annurev.resource.012809.103923

[47] Sumaila UR, Khan AS, Dyck AJ, Watson R, Munro G, Tydemers P, et al A bottom-up re-estimation of global fisheries subsidies. Journal of Bioeconomics. 2010;12(3):201–225. 10.1007/s10818-010-9091-8

[48] Seijo J, Defeo O, Salas S. Fisheries bioeconomics. Theory, modelling and management. FAO Fisheries Technical Paper, Rome, FAO. 1998;(368).

[49] Holley JF, Marchal P. Fishing strategy development under changing conditions: examples from the French offshore fleet fishing in the North Atlantic. ICES Journal of Marine Science. 2004;61(8):1410–1431. 10.1016/j.icesjms.2004.08.010

[50] Vermard Y, Marchal P, Mahévas S, Thébaud O. A dynamic model of the Bay of Biscay pelagic fleet simulating fishing trip choice: the response to the closure of the European anchovy (Engraulis encrasicolus) fishery in 2005. Canadian Journal of Fisheries and Aquatic Sciences. 2008;65(11):2444–2453. 10.1139/F08-147

[51] Rochet MJ, Daurès F, Trenkel VM, Walters C. Capacity management, not stock status or economics, drives fleet dynamics in the Bay of Biscay ecosystem on a decadal time scale. Canadian Journal of Fisheries and Aquatic Sciences. 2012;69(4):695–710. 10.1139/f2012-002

[52] Arreguín-Sánchez F. Catchability: a key parameter for fish stock assessment. Reviews in Fish Biology and Fisheries. 1996;6(2):221–242. 10.1007/BF00182344

[53] Pauly D, Palomares MD. An empirical equation to predict annual increases in fishing efficiency. Fisheries Centre University of British Columbia, Working Paper Series 2010-07. 2010;.

[54] Sethi SA, Branch TA, Watson R. Global fishery development patterns are driven by profit but not trophic level. Proceedings of the National Academy of Sciences. 2010;107(27):12163–12167. 10.1073/pnas.1003236107 PMC2901455 20566867

[55] Lam VWY, Sumaila UR, Dyck A, Pauly D, Watson R. Construction and first applications of a global cost of fishing database. ICES Journal of Marine Science. 2011;68(9):1996–2004. 10.1093/icesjms/fsr121

[56] Millar RB, Fryer RJ. Estimating the size-selection curves of towed gears, traps, nets and hooks. Reviews in Fish Biology and Fisheries. 1999;9(1):89–116. 10.1023/A:1008838220001

[57] Rochet MJ, Collie JS, Jennings S, Hall SJ. Does selective fishing conserve community biodiversity? Predictions from a length-based multispecies model. Journal of the Fisheries Board of Canada. 2011;68(3):469–486.

[58] Law R, Plank MJ, Kolding J. On balanced exploitation of marine ecosystems: results from dynamic size spectra. ICES Journal of Marine Science. 2012;69(4):602–614. 10.1093/icesjms/fss031

[59] Garcia SM, Kolding J, Rice J, Rochet MJ, Zhou S, Arimoto T, et al Reconsidering the Consequences of Selective Fisheries. Science. 2012;335(6072):1045–1047. 10.1126/science.1214594 22383833

[60] Watson R, Revenga C, Kura Y. Fishing gear associated with global marine catches. Fisheries Research. 2006;79(1-2):97–102. 10.1016/j.fishres.2006.01.013

[61] Pauly D. The Sea Around Us Project: Documenting and Communicating Global Fisheries Impacts on Marine Ecosystems. AMBIO: A Journal of the Human Environment. 2007;36(4):290–295. 10.1579/0044-7447(2007)36[290:TSAUPD]2.0.CO;2 17626465

[62] Froese R, Thorson JT, Reyes RB Jr. A Bayesian approach for estimating length-weight relationships in fishes. Journal of Applied Ichthyology. 2013;30(1):78–85. 10.1111/jai.12299

[63] Ricard D, Minto C, Jensen OP, Baum JK. Examining the knowledge base and status of commercially exploited marine species with the RAM Legacy Stock Assessment Database. Fish and Fisheries. 2011;13(4):380–398. 10.1111/j.1467-2979.2011.00435.x

[64] Hilborn R, Branch TA, Ernst B, Magnusson A, Minte-Vera CV, Scheuerell MD, et al STATE OF THE WORLD’S FISHERIES. Annual Review of Environment and Resources. 2003;28(1):359–399. 10.1146/annurev.energy.28.050302.105509

[65] Piroddi C, Gristina M, Zylich K, Greer K, Ulman A, Zeller D, et al Reconstruction of Italy’s marine fisheries removals and fishing capacity, 1950–2010. Fisheries Research. 2015;172:137–147. 10.1016/j.fishres.2015.06.028

[66] Robert CP, Casella G. Monte Carlo Statistical Methods. New York, NY, USA: Dordrecht: Springer Science+Business Media New York; 2004 10.1007/978-1-4757-4145-2

[67] Friedland KD, Stock C, Drinkwater KF, Link JS, Leaf RT, Shank BV, et al Pathways between Primary Production and Fisheries Yields of Large Marine Ecosystems. PLoS ONE. 2012;7(1):e28945 10.1371/journal.pone.0028945 22276100 PMC3262787

[68] Gregg WW, Casey NW. Sampling biases in MODIS and SeaWiFS ocean chlorophyll data. Remote Sensing of Environment. 2007;111(1):25–35. 10.1016/j.rse.2007.03.008

[69] Johnson R, Strutton PG, Wright SW, McMinn A, Meiners KM. Three improved satellite chlorophyll algorithms for the Southern Ocean. Journal Of Geophysical Research-Oceans. 2013;118:1–10. 10.1002/jgrc.20270

[70] Cressey D. Fisheries: eyes on the ocean. Nature. 2015;519:280–281. 25788078 10.1038/519280a

[71] Pauly D, Zeller D. Catch reconstructions reveal that global marine fisheries catches are higher than reported and declining. Nature Communications. 2016;7:10244 10.1038/ncomms10244 26784963 PMC4735634

[72] Chassot E, Bonhommeau S, Dulvy N. Global marine primary production constrains fisheries catches. Ecology Letters. 2010;13:495–505. 10.1111/j.1461-0248.2010.01443.x 20141525

[73] Blanchard JL, Jennings S, Law R, Castle MD, McCloghrie P, Rochet MJ, et al How does abundance scale with body size in coupled size-structured food webs? Journal of Animal Ecology. 2009;78(1):270–280. 10.1111/j.1365-2656.2008.01466.x 19120607

[74] Myers RA. Stock and recruitment: generalizations about maximum reproductive rate, density dependence, and variability using meta-analytic approaches. ICES Journal of Marine Science. 2001;58(5):937–951. 10.1006/jmsc.2001.1109

[75] Conover WJ. Practical Nonparametric Statistics, Third Edition New York, NY, USA: John Wiley & Sons, Inc.; 1999.

[76] Kooijmann SALM. Dynamic Energy Mass Budgets in Biological Systems. Cambridge: Cambridge University Press; 2000.

[77] Clarke A, Johnston NM. Scaling of metabolic rate with body mass and temperature in teleost fish. Journal of Animal Ecology. 1999;68(5):893–905. 10.1046/j.1365-2656.1999.00337.x

[78] Rall BC, Brose U, Hartvig M, Kalinkat G, Schwarzmuller F, Vucic-Pestic O, et al Universal temperature and body-mass scaling of feeding rates. Philosophical Transactions of the Royal Society B: Biological Sciences. 2012;367(1605):2923–2934. 10.1098/rstb.2012.0242 PMC3479751 23007080

[79] Bopp L, Resplandy L, Orr JC, Doney SC, Dunne JP, Gehlen M, et al Multiple stressors of ocean ecosystems in the 21st century: projections with CMIP5 models. Biogeosciences. 2013;10(10):6225–6245. 10.5194/bg-10-6225-2013

