# Supplementary figures and images for: Formulation, General Features and Global Calibration of a Bioenergetically-Constrained Fishery Model
Source: PLoS One. 2017 Jan 19;12(1):e0169763. doi: 10.1371/journal.pone.0169763 (PMC5245811; doi:10.1371/journal.pone.0169763)

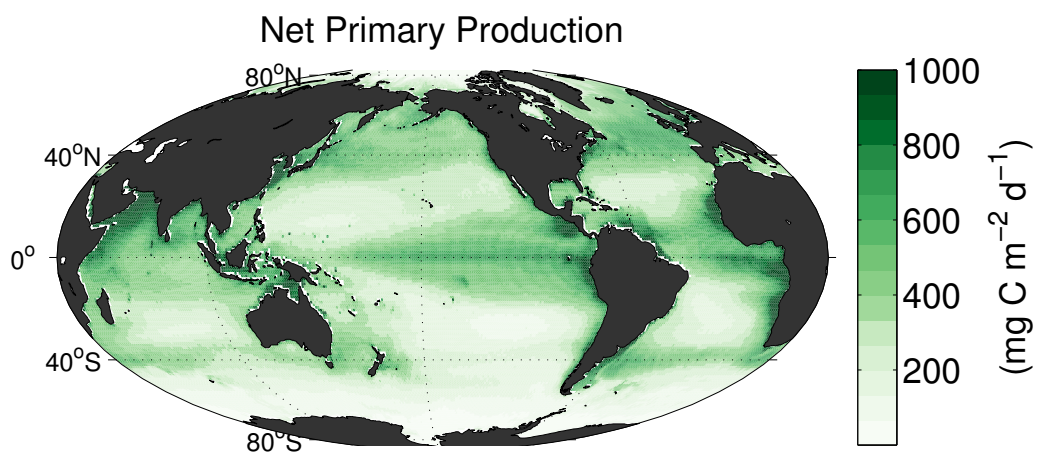

S1 Fig. Annual average net primary production (mg C m<sup>-2</sup> d<sup>-1</sup>) forcing applied in BOATS.

Supplement: S1 Fig — (PDF) [file pone.0169763.s001.pdf]

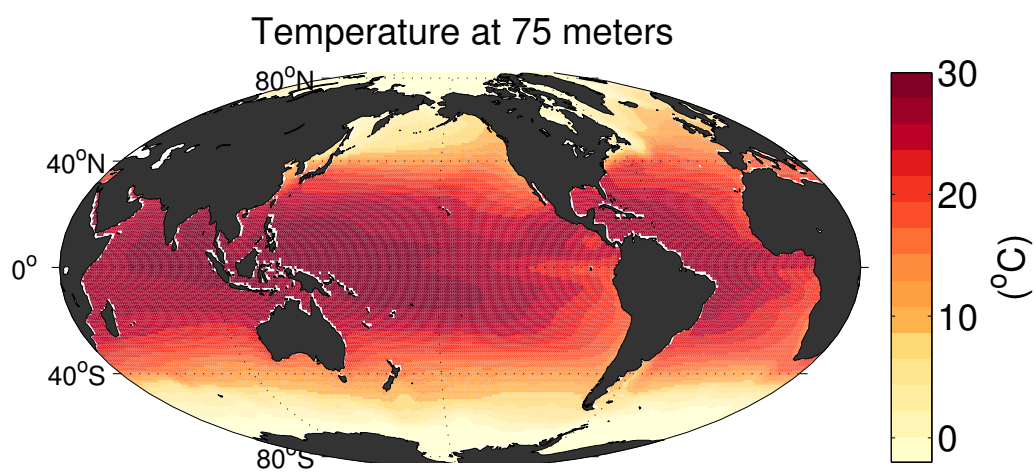

S2 Fig. Annual average 75-meter average temperature (°C) forcing applied in BOATS.

Supplement: S2 Fig — (PDF) [file pone.0169763.s002.pdf]

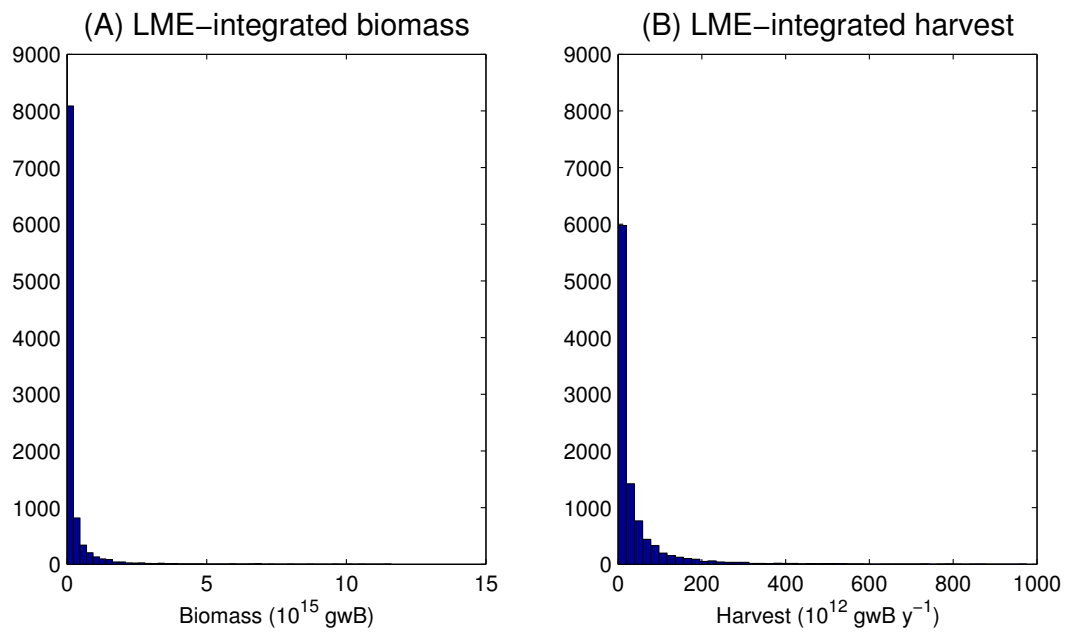

**S4 Fig.** Histograms of LME-integrated (A) biomass and (B) harvest from the Monte Carlo suite.

Supplement: S4 Fig — (PDF) [file pone.0169763.s004.pdf]
