# Supplementary material for: Formulation, General Features and Global Calibration of a Bioenergetically-Constrained Fishery Model
Source: PLoS One. 2017 Jan 19;12(1):e0169763. doi: 10.1371/journal.pone.0169763 (PMC5245811; doi:10.1371/journal.pone.0169763)
Supplement: S3 Fig — (PDF) [file pone.0169763.s003.pdf]

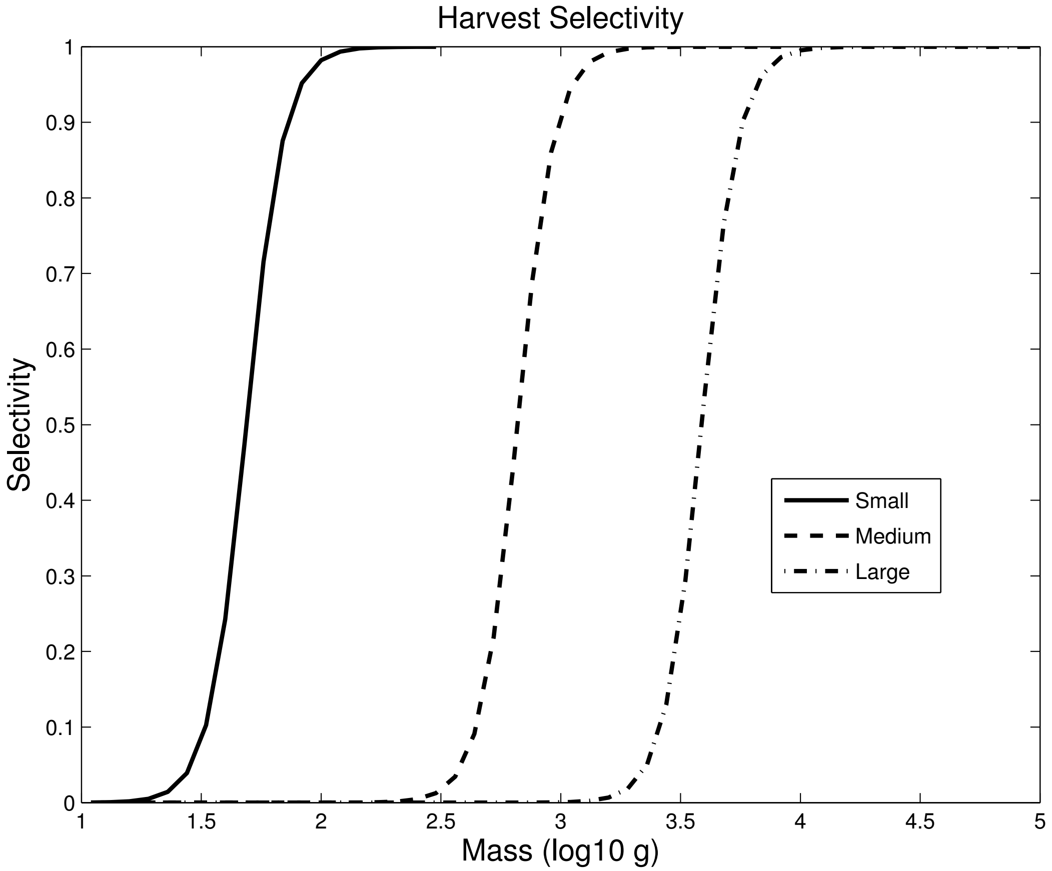

S3 Fig. Harvest selectivity σ_k by group. Solid, dashed, and dot-dashed curves are small, medium, and large group harvest.
