# Supplementary material for: Formulation, General Features and Global Calibration of a Bioenergetically-Constrained Fishery Model
Source: PLoS One. 2017 Jan 19;12(1):e0169763. doi: 10.1371/journal.pone.0169763 (PMC5245811; doi:10.1371/journal.pone.0169763)
Supplement: S5 Fig — (PDF) [file pone.0169763.s005.pdf]

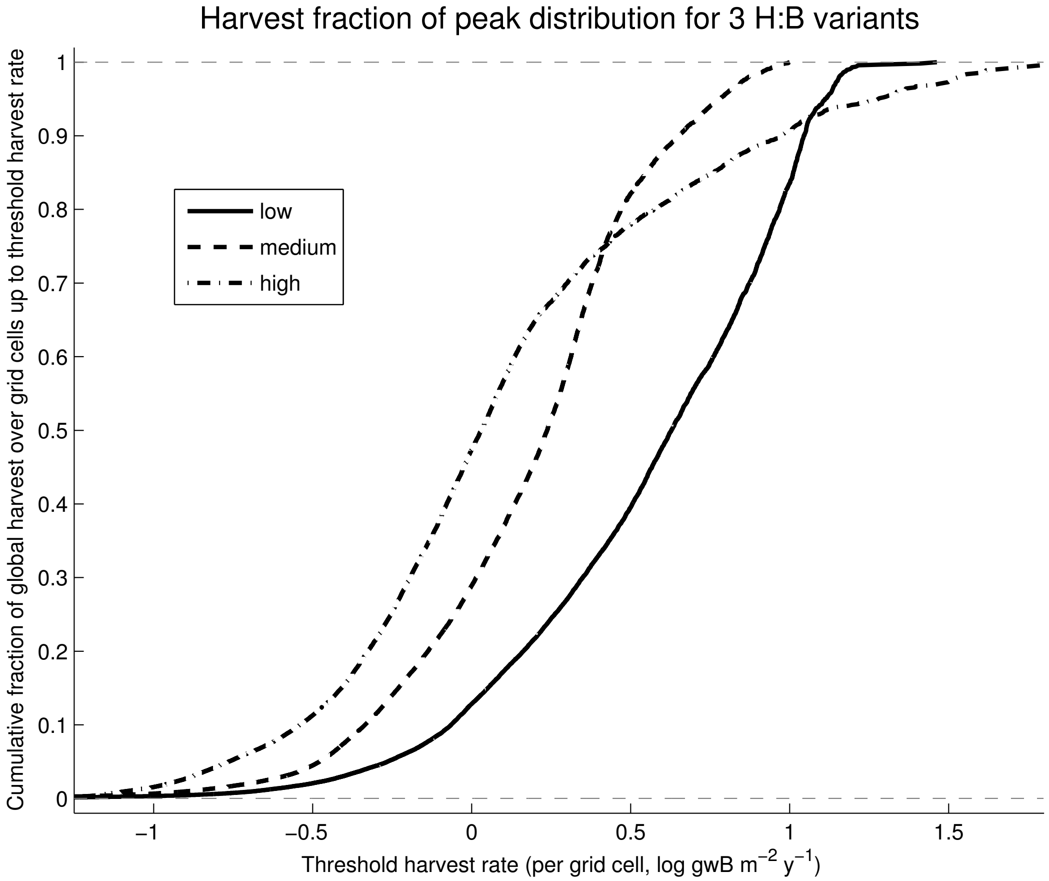

S5 Fig. Cumulative fraction of global harvest integrated over all grid cells with a harvest rate less than a threshold harvest rate for low, medium, and high harvest to biomass ratio (H:B) model variants. Solid, dashed, and dot-dashed curves represent low, medium, and high H:B model variants. Curves are from the annual average of the year of peak harvest, and correspond to Fig 10A-C.
