# Supplementary material for: Formulation, General Features and Global Calibration of a Bioenergetically-Constrained Fishery Model
Source: PLoS One. 2017 Jan 19;12(1):e0169763. doi: 10.1371/journal.pone.0169763 (PMC5245811; doi:10.1371/journal.pone.0169763)
Supplement: S1 Table — (PDF) [file pone.0169763.s006.pdf]

S1 Table. Ecological model parameters. The † symbol in the parameter column identifies parameters whose probability distributions were included in the Monte Carlo suite.

**Parameter**	**Name**	**Value [Range]**	**Unit**
m_0	Lower bound of smallest mass class	10	g
m_u	Upper bound of largest mass class	100 000	g
N_M	Number of mass classes	50	-
$m_{i,L}$	Mass at lower bound of mass class i	-	g
m_i	Representative mass of a mass class i	-	g
$m_{\infty,k}$	Asymptotic mass of group k	(314 8500 100000)	g
T_r	Reference temperature for $a(T)$	10	°C
k_B	Boltzmann's constant	8.617×10^{-5}	eV K^{-1}
† $\omega_{a,A}$	Growth activation energy of metabolism	0.3116 [0.45 ± 0.09]	eV
† $\omega_{a,\lambda}$	Mortality activation energy of metabolism	0.3756 [0.45 ± 0.09]	eV
† b	Growth allometric scaling exponent	0.6787 [0.7 ± 0.05]	Unitless
† A_0	Allometric growth constant	3.6633 [4.46 ± 0.5]	g^{1-b} s^{-1}
ϵ_a	Activity fraction	0.8	Unitless
c_s	Slope of $s_k(m)$	5	Unitless
η	Ratio of mature to asymptotic mass	0.25 [0.25 ± 0.075]	Unitless
† α	Trophic efficiency	0.16 [0.1,0.16]	Unitless
† β	Predator to prey mass ratio	7609 [850,10000]	Unitless
τ	Trophic scaling	-0.2047	Unitless
m_L	Mass of large phytoplankton	4×10^{-6}	g
m_S	Mass of small phytoplankton	4×10^{-15}	g
† k_E	Eppley constant for phytoplankton growth	0.0667 [0.0631 ± 0.009]	$°C^{-1}$
P^*	Pivotal phytoplankton concentration	1.9 ± 0.3	mmol C m^{-3}
† Π^*	NPP referenced to $T_C = 0°C$	0.3135 [0.37 ± 0.1]	mmol C m^{-3} d^{-1}
† ζ_1	Mortality constant	0.2701 [0.55 ± 0.57]	Unitless
† h	Allometric mortality scaling exponent	0.4641 [0.54 ± 0.09]	Unitless
ϕ_f	Fraction of females	0.5	Unitless
$\phi_{\Pi_\Psi,C}$	Fraction of NPP to commercial fish groups	1	Unitless
$\phi_{C,k}$	Fraction of $\phi_{\Pi_\Psi,C}$ allocated to a group k	1/3	Unitless
† s_e	Egg to recruit survival fraction	0.0327 [$10^{-3.5}$, 0.5]	Unitless
m_e	Egg mass	5.2×10^{-4}	g
