# Supplementary material for: Formulation, General Features and Global Calibration of a Bioenergetically-Constrained Fishery Model
Source: PLoS One. 2017 Jan 19;12(1):e0169763. doi: 10.1371/journal.pone.0169763 (PMC5245811; doi:10.1371/journal.pone.0169763)
Supplement: S2 Table — (PDF) [file pone.0169763.s007.pdf]

S2 Table. Gear types in terms of selectivity functional form (S = sigmoidal, G = gaussian, O = other) and percentage of global harvest (Table 3 of Lam et al., 2011).

**Gear Type**	**Selectivity**	**Percentage of global harvest**
Seine	S	29
Gillnet	G	21
Midwater trawl	S	16
Bottom trawl	S	11
Hook and line	G	8
Longline tuna	G	3
Shrimp trawl	S	3
Trap	S	2
Net	S	2
Dredge	S	2
Pole and line tuna	G	1
Purse-seine tuna	S	1
Hand	G	1
Spear	G	< 0.5
Castnet	S	< 0.5
Liftnet	S	< 0.5
Trammelnet	S	< 0.5
Bomb/chemical	O	< 0.5

References

Lam VWY, Sumaila UR, Dyck A, Pauly D, Watson R. Construction and first applications of a global cost of fishing database. ICES Journal of Marine Science. 2011; 68(9):1996-2004. doi:10.1093/icesjms/fsr121.
