# Supplementary material for: Formulation, General Features and Global Calibration of a Bioenergetically-Constrained Fishery Model
Source: PLoS One. 2017 Jan 19;12(1):e0169763. doi: 10.1371/journal.pone.0169763 (PMC5245811; doi:10.1371/journal.pone.0169763)
Supplement: S3 Table — (PDF) [file pone.0169763.s008.pdf]

S3 Table. Large Marine Ecosystem numbers and names. LMEs with a * before the number are ignored in the analysis since they are high latitude sites (and so NPP is not reliable) or inland seas.

**LME**	**Name**	**LME**	**Name**
1	Baltic Sea	34	Indonesian Sea
2	Sea of Okhotsk	35	East Brazil Shelf
3	North Sea	36	Humboldt Current
4	Gulf of Alaska	37	Benguela Current
5	Labrador - Newfoundland	38	North Australian Shelf
6	Celtic-Biscay Shelf	39	Northeast Australian Shelf
7	Sea of Japan	40	Agulhas Current
8	Oyashio Current	41	Northwest Australian Shelf
9	Scotian Shelf	42	East Central Australian Shelf
10	California Current	43	South Brazil Shelf
*11	Black Sea	44	New Zealand Shelf
12	Northeast U.S. Continental Shelf	45	Patagonian Shelf
13	Mediterranean Sea	46	Southeast Australian Shelf
14	Iberian Coastal	*47	Antarctica
15	Kuroshio Current	48	South West Australian Shelf
16	Yellow Sea	49	West Central Australian Shelf
17	Canary Current	50	Faroe Plateau
18	East China Sea	51	Iceland Shelf and Sea
19	Southeast U.S. Continental Shelf	52	Greenland Sea
20	Gulf of California	53	Norwegian Sea
21	Gulf of Mexico	54	Barents Sea
22	Arabian Sea	*55	Kara Sea
23	Red Sea	*56	Laptev Sea
24	Insular Pacific-Hawaiian	*57	East Siberian Sea
25	Caribbean Sea	58	West Bering Sea
26	South China Sea	59	Aleutian Islands
27	Bay of Bengal	60	East Bering Sea
28	Pacific Central-American Coastal	*61	Canadian Eastern Arctic - West Greenland
29	Sulu-Celebes Sea	*62	Hudson Bay Complex
30	Gulf of Thailand	*63	Beaufort Sea
31	North Brazil Shelf	*64	Canadian High Arctic - North Greenland
32	Guinea Current	*65	Central Arctic
33	Somali Coastal Current	*66	Northern Bering - Chukchi Seas
