# Supplementary material for: Formulation, General Features and Global Calibration of a Bioenergetically-Constrained Fishery Model
Source: PLoS One. 2017 Jan 19;12(1):e0169763. doi: 10.1371/journal.pone.0169763 (PMC5245811; doi:10.1371/journal.pone.0169763)
Supplement: S4 Table — (PDF) [file pone.0169763.s009.pdf]

S4 Table. Partitioning of SAUP functional groups into small (S), medium (M), large (L), and other (O) size groups.

**Functional Group**	**Size Group**
Large benthopelagics	L
Medium demersals	M
Small pelagics	S
Small to medium flatfishes	O
Medium pelagics	M
Large demersals	L
Other demersal invertebrates	O
Lobsters and crabs	S
Shrimps	S
Cephalopods	O
Small to medium rays	O
Small demersals	S
Large flatfishes	L
Medium benthopelagics	M
Other groups	O
Small bathydemersals	S
Large bathydemersals	L
Large pelagics	L
Large sharks	L
Medium reef association fish	M
Large reef association fish	L
Small reef association fish	S
Large rays	L
Medium bathydemersals	M
Medium bathypelagics	M
Small bathypelagics	S
Small to medium sharks	O
Krill	S
